# Supplementary material for: Deep learning enables satellite-based monitoring of large populations of terrestrial mammals across heterogeneous landscape
Source: Nat Commun. 2023 May 27;14:3072. doi: 10.1038/s41467-023-38901-y (PMC10224963; doi:10.1038/s41467-023-38901-y)
Supplement: Supplementary file 1 — Supplementary information [file 41467_2023_38901_MOESM1_ESM.pdf]

Supplementary Information for

Deep learning enables satellite-based monitoring of large populations of terrestrial mammals across heterogeneous landscapes

Zijing Wu, Ce Zhang, Xiaowei Gu, Isla Duporge, Lacey F. Hughey, Jared A. Stabach, Andrew K. Skidmore, J. Grant C. Hopcraft, Stephen J. Lee, Peter M. Atkinson, Douglas J. McCauley, Richard Lamprey, Shadrack Ngene, Tiejun Wang*

*Corresponding author. Email: t.wang@utwente.nl (T.W.)

This PDF file includes:

Supplementary Fig. 1 to 10
Supplementary Table 1 to 5
Supplementary Equation (1)

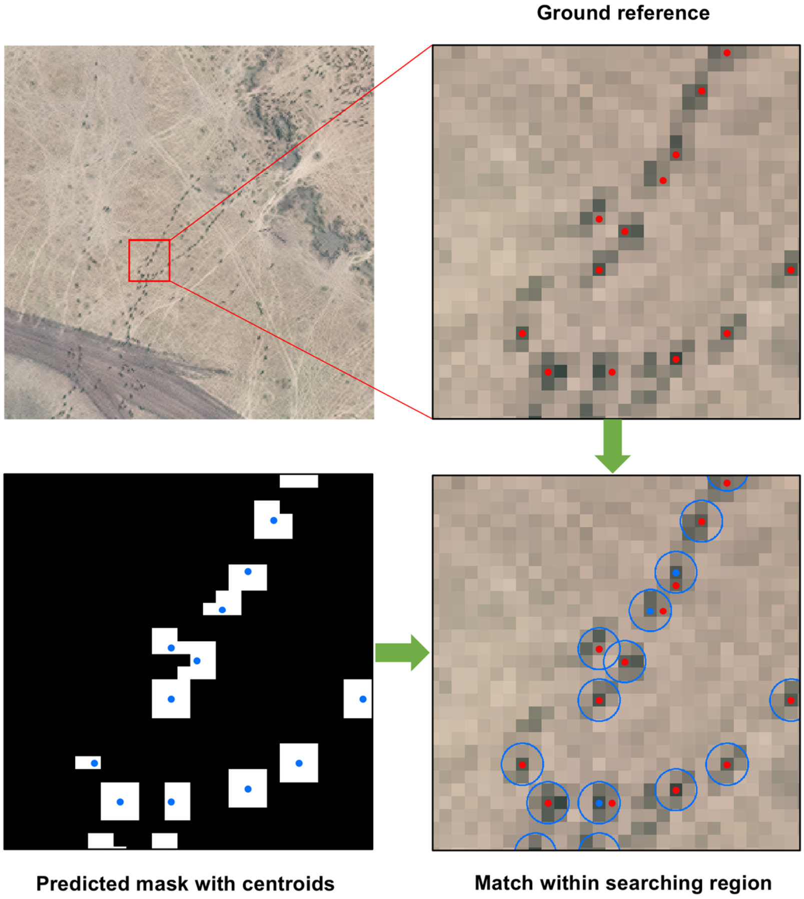

Supplementary Fig. 1.

Model evaluation at the level of wildebeest individuals. The prediction mask produced by the U-Net model was converted to wildebeest centroids using *K*-Means clustering, which represent the predicted wildebeest points. Then these points were matched with the ground reference points using a search buffer, shown as the blue circles in the figure. When the ground reference point falls within the searching distance, the predicted point will be considered a correct prediction. Satellite image © 2009 Maxar Technologies.

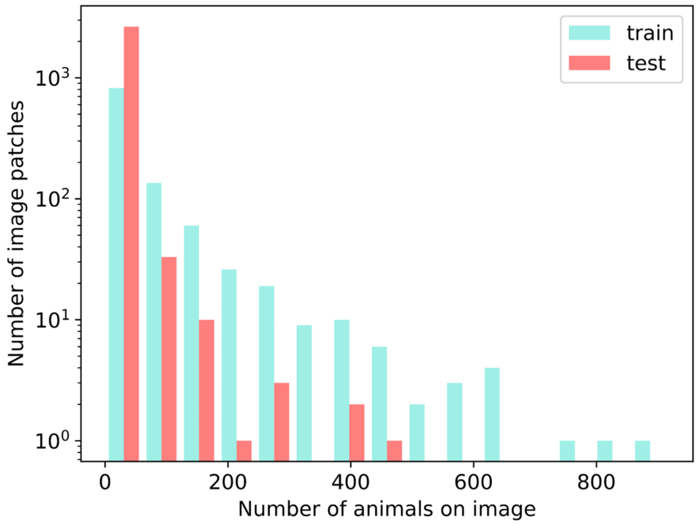

Supplementary Fig. 2.

Distribution of the number of animals on each image in the training and test dataset. The validation data is randomly selected from the training dataset (10% of it) and thus is not shown in this figure.

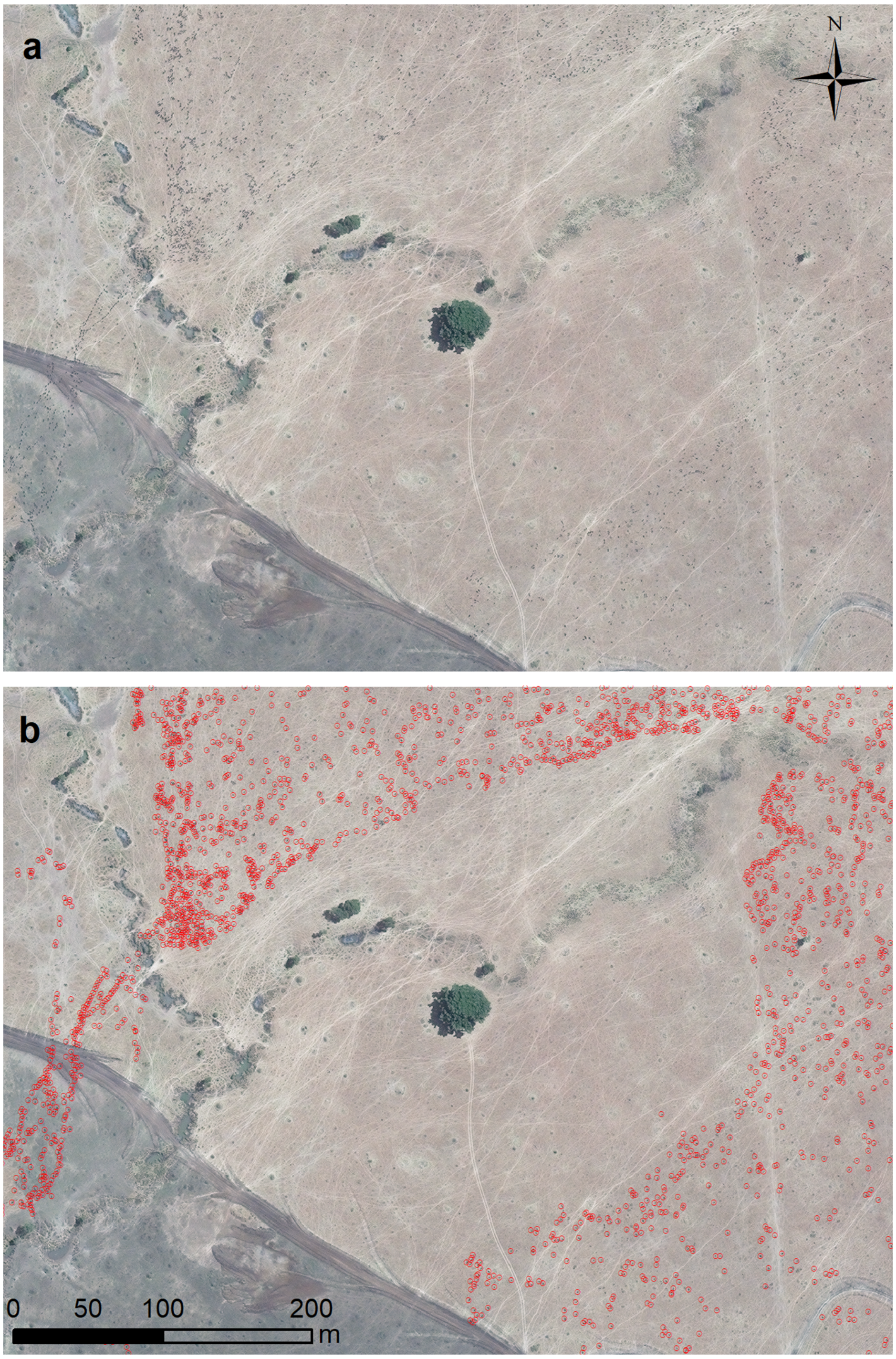

Supplementary Fig. 3.

Example of wildebeest detection in 2009. **a** The original satellite image. **b** The detection results on the satellite image. The detected wildebeests are represented by red circles. In this example, wildebeest congregate while avoiding the large tree in the center where predators sometimes refuge and form a pattern like a vacuole. Satellite image © 2009 Maxar Technologies.

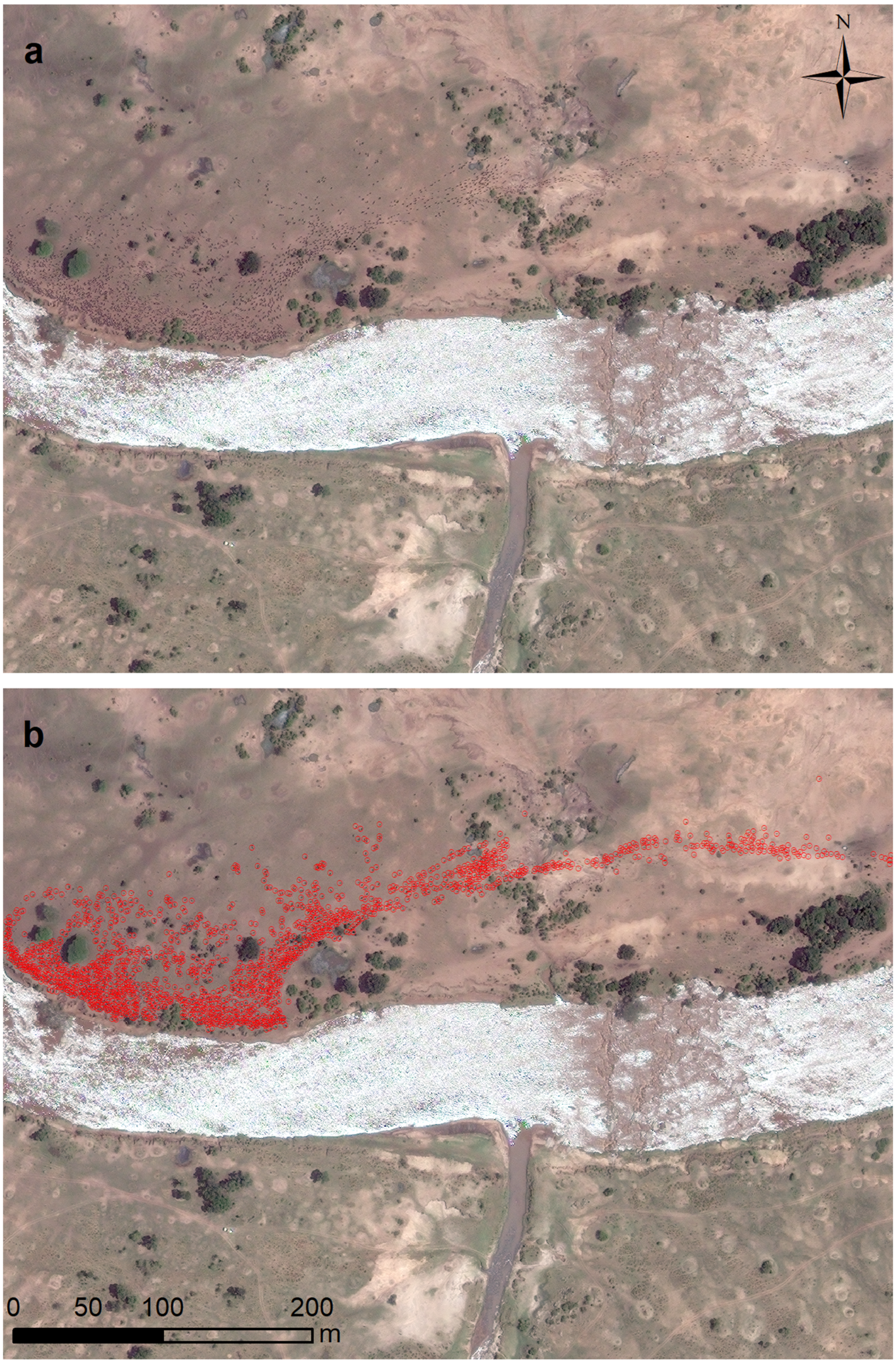

Supplementary Fig. 4.

Example of wildebeest detection in a mixed savanna-woodland in 2010. a The original satellite image. **b** The detection results on the satellite image. The detected wildebeest are represented by the red circles. In this example, the wildebeest are massing along the bank of the Mara River ahead of crossing the river. Satellite image © 2010 Maxar Technologies.

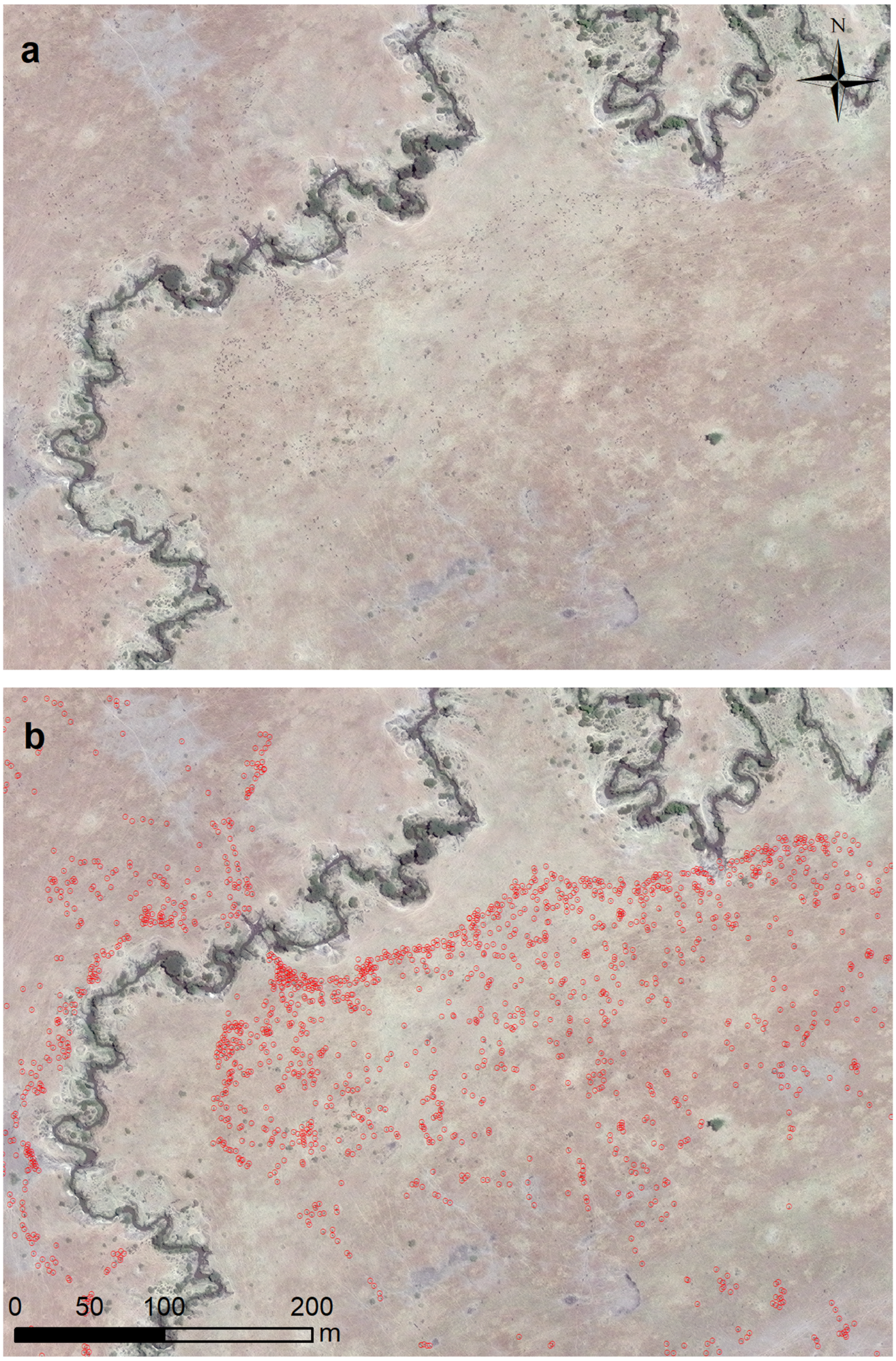

Supplementary Fig. 5.

Example of wildebeest crossing the river in grassland in 2013. a The original satellite image. **b** The detection results on the satellite image. The detected wildebeest are represented by the red circles. In this example, the wildebeest form a wavefront pattern as has been previously described at smaller scales for this species using aerial imagery. Satellite image © 2013 Maxar Technologies.

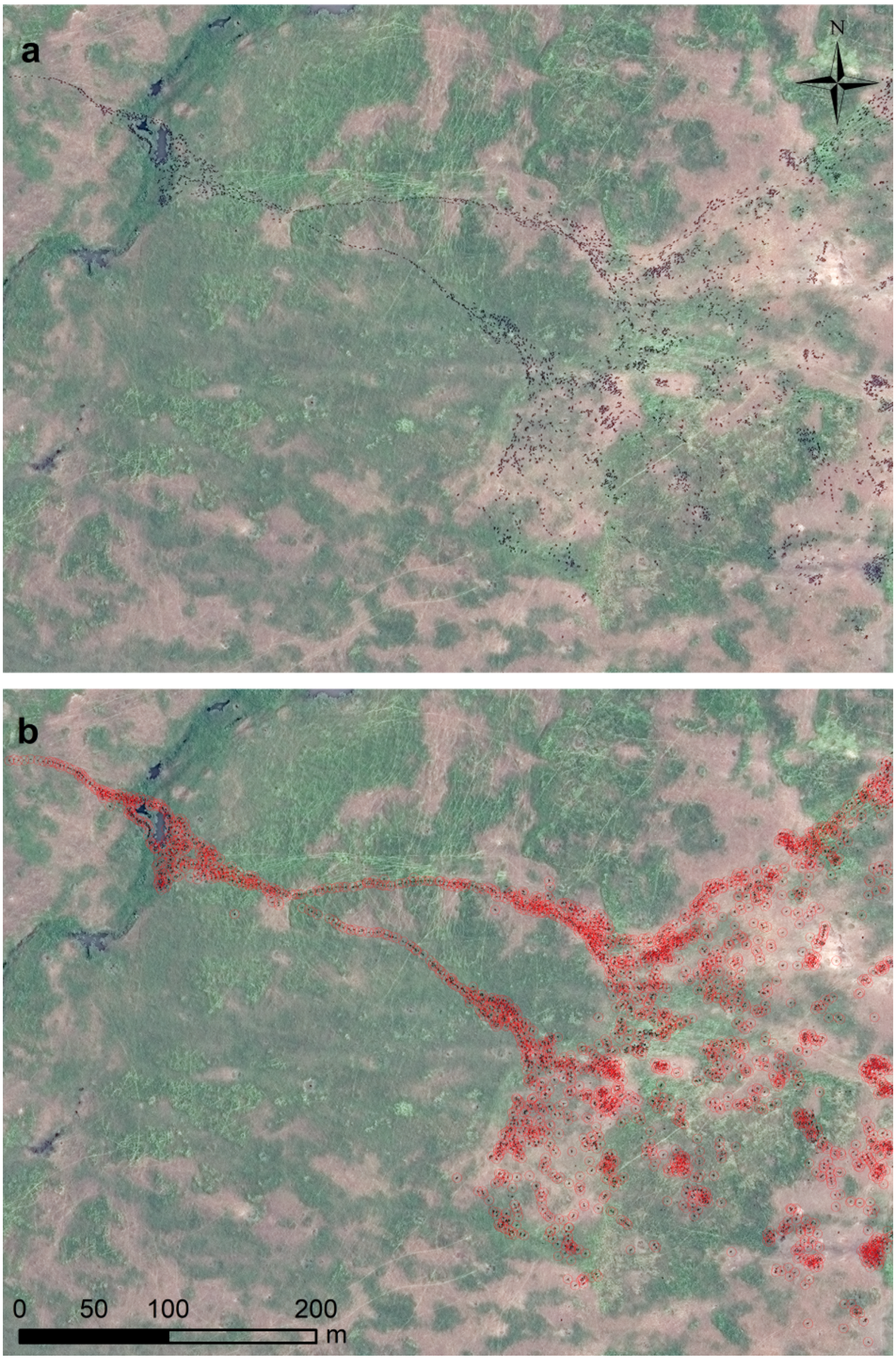

Supplementary Fig. 6.

Example of wildebeest detection near the river in 2015. a The original satellite image. **b** The detection results on the satellite image. The detected wildebeest are represented by the red circles. In this example, the wildebeest form linear and wavefront patterns. They are likely heading towards the river, grouping together and staging to cross the river. Satellite image © 2015 Maxar Technologies.

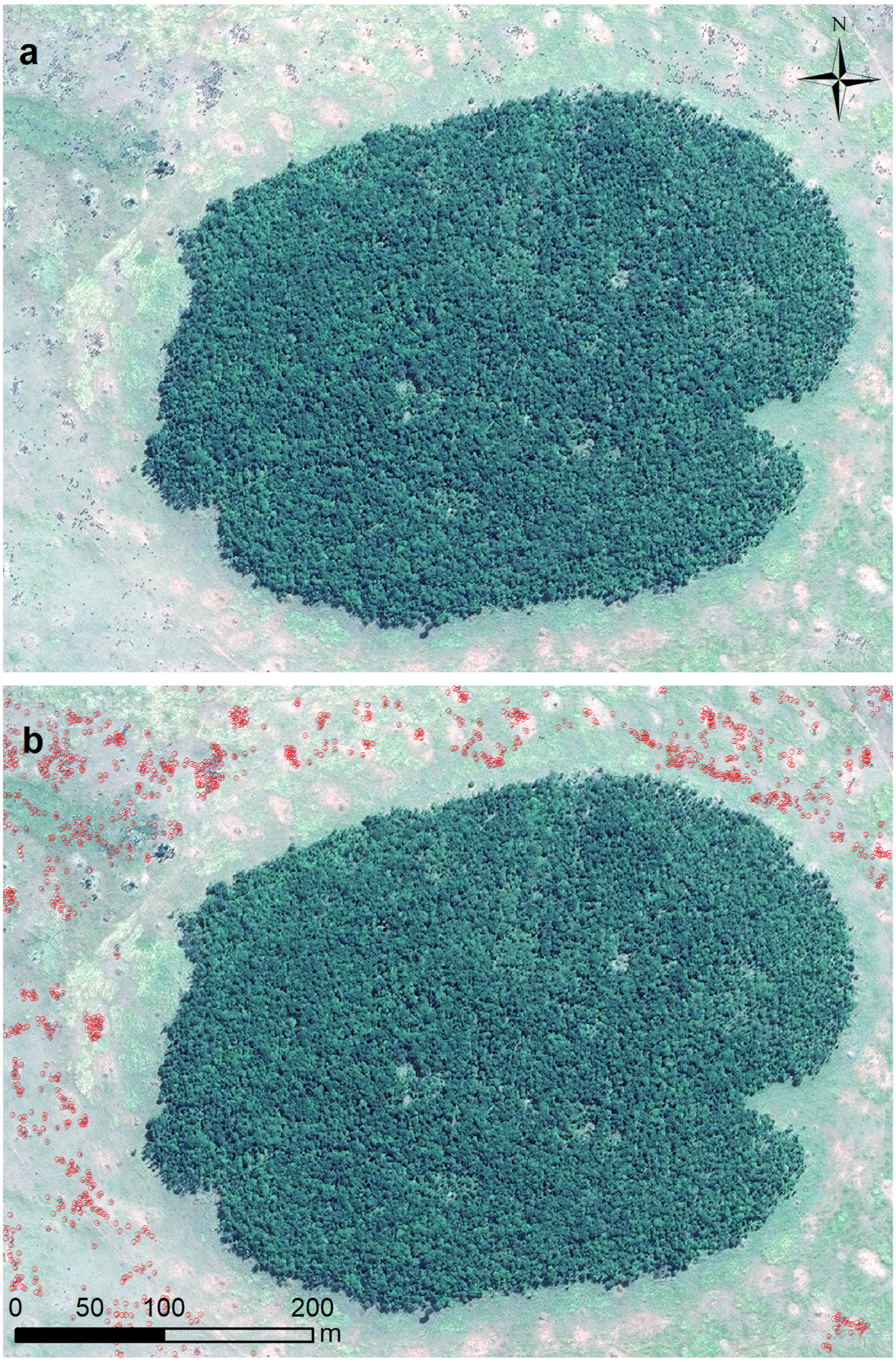

Supplementary Fig. 7.

Example of wildebeest detection in grassland in 2018. a The original satellite image. **b** The detection results on the satellite image. The detected wildebeest are represented by the red circles. In this example, the wildebeest form a dispersed pattern around the base of the steep hill capped with thickets. Satellite image © 2018 Maxar Technologies.

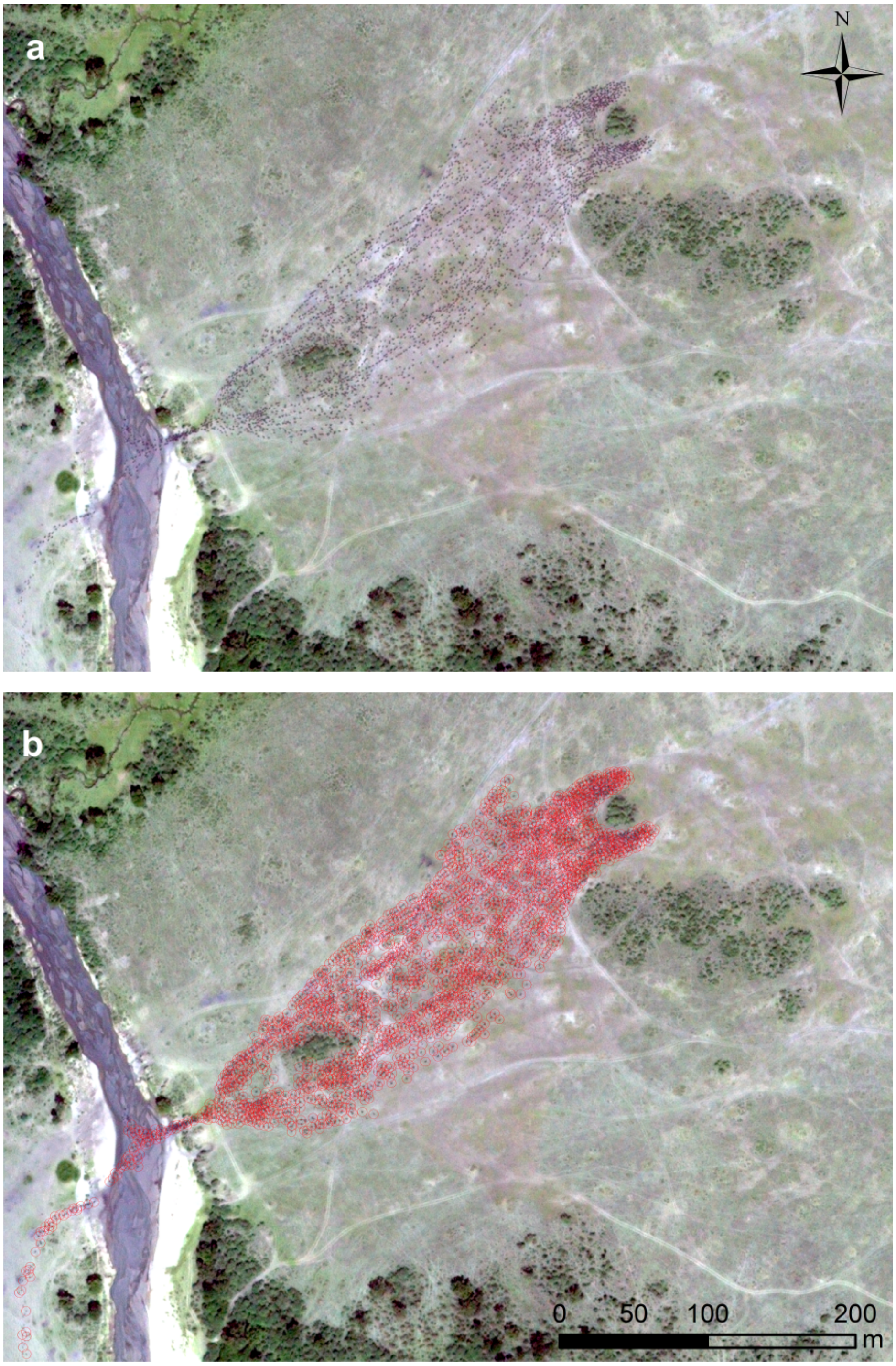

Supplementary Fig. 8.

Example of wildebeests crossing the river in 2020. a The original satellite image. **b** The detection results on the satellite image. The detected wildebeest are represented by the red circles. In this example, the wildebeest are migrating forming a queue to cross the river, also forming vacuoles around the trees. Satellite image © 2020 Maxar Technologies.

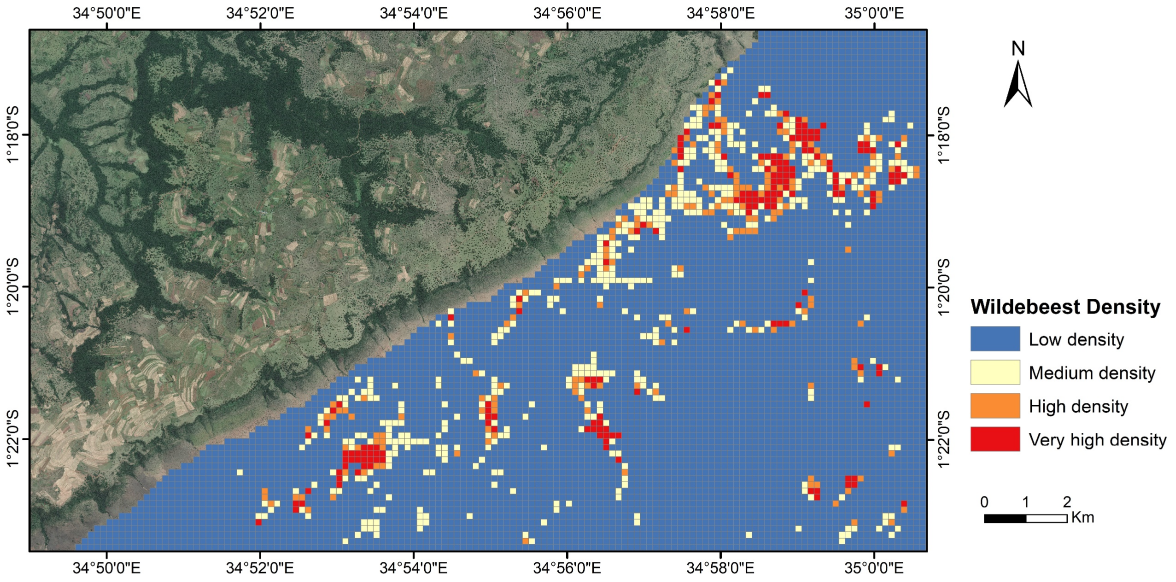

Supplementary Fig. 9.

The wildebeest density map in 2009 used for testing dataset sampling using a stratified random sampling approach. The density data were generated from the preliminary model. All the grids were classified using the standard deviation method according to the wildebeest count, and they were grouped into 4 density categories: low density (0-42 animals), medium density (43-89 animals), high density (90-135 animals), and very high density (136-526 animals), and the percentage of each category is 86%, 8%, 3%, and 3%, respectively. 100 testing sample grids were randomly selected within each category separately according to the proportion. The northwestern area on the map where there are no density data is the ranch and cultivation area above the Siria Escarpment; wildebeest are normally absent from this area, which is outside the Mara Triangle Conservancy. Satellite image © 2009 Maxar Technologies.

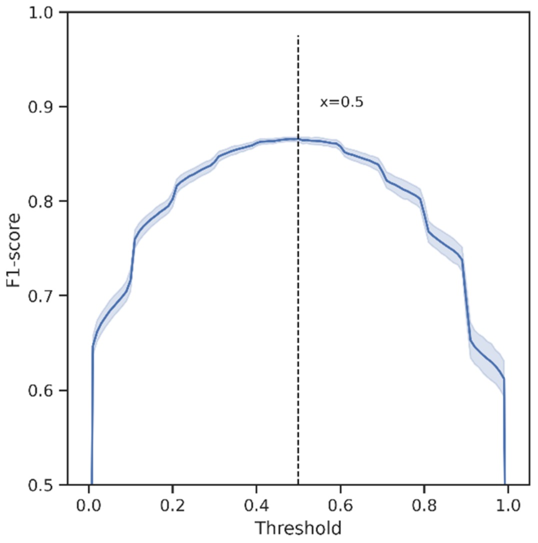

Supplementary Fig. 10.

F1-score of the U-Net model under different thresholds. The model was trained 10 times separately, and the line plot aggregates multiple F1-score values of 10 models at each threshold. The solid line represents the average values of all the 10 iterations. The grey area is the 95% confidence interval of the F1-score. This analysis was conducted using the dataset of 2009.

Supplementary Table 1.
The level of agreement across the four annotators during wildebeest labeling

Satellite Image	Wildebeest labels agreed by four annotators	Wildebeest labels agreed by only three annotators	Wildebeest labels agreed after re-examination
2009	20770 (94.1%)	1142 (5.2%)	153 (0.7%)
2010	8012 (98.0%)	159 (1.9%)	8 (0.1%)
2013	10284 (96.5%)	351 (3.3%)	24 (0.2%)
2015	621 (97.3%)	17 (2.7%)	0 (0.0%)
2018	10775 (95.4%)	414 (3.7%)	109 (0.9%)
2020	12419 (98.1%)	236 (1.9%)	6 (0.0%)
Overall	62881 (96.1%)	2319 (3.5%)	300 (0.4%)

Supplementary Table 2.

The satellite images used for model training, testing, and wildebeest counting. Each patch represents of 336 × 336 pixels.

Satellite	Acquisition date	Spatial resolution	Training dataset size (patch)	Wildebeest labels in training set	Test dataset size (patch)	Wildebeest labels in test set
GE01	11/Aug/2009	43 cm	191	19,816	100	2,249
GE01	24/Sep/2010	44 cm	130	7,120	100	1,059
GE01	10/Aug/2013	45~50 cm	106	9,440	200	1,219
WV03	17/Jul/2015	38 cm	12	0	200	638
GE01	02/Aug/2018	42~48 cm	135	10,861	200	437
WV02	08/Oct/2020	50 cm	523	6,669	1900	5,992

Supplementary Table 3.
The testing performance of the wildebeest detection model.

Image acquisition date	Spatial resolution	Precision	Recall	F1-score
11/Aug/2009	43 cm	90.72%	83.76%	87.09%
24/Sep/2010	44 cm	97.80%	75.58%	85.27%
10/Aug/2013	45~50 cm	82.68%	78.26%	80.40%
17/Jul/2015	38 cm	96.34%	87.52%	91.70%
02/Aug/2018	42~48 cm	91.40%	74.00%	81.76%
08/Oct/2020	50 cm	85.40%	82.97%	84.17%
Overall	38~50 cm	87.85%	81.86%	84.75%

Supplementary Table 4.
Accuracy of individual base models compared against the accuracy of ensemble model.

Model	Precision	Recall	F1-score	AUC
Base 1	79.04%	78.81%	78.92%	0.80
Base 2	79.13%	76.67%	77.88%	0.79
Base 3	79.68%	76.87%	78.25%	0.79
Base 4	79.21%	77.97%	78.58%	0.79
Base 5	81.38%	68.56%	74.42%	0.76
Base 6	78.85%	77.22%	78.03%	0.79
Base 7	78.39%	80.99%	79.67%	0.80
Base 8	79.00%	79.93%	79.46%	0.80
Base 9	82.50%	74.33%	78.20%	0.79
Base 10	77.58%	80.05%	78.80%	0.79
Ensemble	87.85%	81.86%	84.75%	0.88

Supplementary Table 5.

Area Under the Curve (AUC) of the wildebeest detection model trained with different weights of Tversky loss. AUC is the area under the precision-recall curve, and a higher AUC indicates a better performance. The weight in the legend is the weight for false positives, β. The model achieves the best performance when the weight is 0.9 (see text in bold).

Weight in Tversky loss	AUC
0.01	0.66
0.1	0.75
0.2	0.80
0.3	0.86
0.4	0.89
0.5	0.90
0.6	0.91
0.7	0.92
0.8	0.93
**0.9**	**0.94**
0.99	0.89

Supplementary Equation (1)

$$T(\alpha,\beta) = 1 - \frac{\sum_{i=1}^{N} p_i g_i + \epsilon}{\sum_{i=1}^{N} p_i g_i + \alpha \sum_{i=1}^{N}(1-p_i)g_i + \beta \sum_{i=1}^{N} p_i(1-g_i) + \epsilon} \quad (1)$$

Where:
p_i denotes the predicted probability of a pixel i to be a wildebeest pixel;
g_i denotes the ground reference value, which is 1 for a wildebeest pixel and 0 for a non-wildebeest pixel;
$\sum_{i=1}^{N} p_i g_i$ summarizes the wildebeest true positives;
$\sum_{i=1}^{N}(1-p_i)g_i$ summarizes the false negatives;
$\sum_{i=1}^{N} p_i(1-g_i)$ summarizes the false positives;
α is the weight of penalties for false negatives;
β is the weight of penalties for false positives,
the sum of α and β is 1.
