# Supplementary material for: Deep learning enables satellite-based monitoring of large populations of terrestrial mammals across heterogeneous landscape
Source: Nat Commun. 2023 May 27;14:3072. doi: 10.1038/s41467-023-38901-y (PMC10224963; doi:10.1038/s41467-023-38901-y)
Supplement: Supplementary file 3 — Description of Additional Supplementary Files [file 41467_2023_38901_MOESM3_ESM.pdf]

Description of Additional Supplementary Files

File Name: Supplementary Data 1

Description: The metadata of satellite images used in the study

File Name: Supplementary Data 2

Description: A Microsoft PowerPoint presentation showcasing 25 animated GIFs
