# Supplementary material for: Deep learning enables satellite-based monitoring of large populations of terrestrial mammals across heterogeneous landscape
Source: Nat Commun. 2023 May 27;14:3072. doi: 10.1038/s41467-023-38901-y (PMC10224963; doi:10.1038/s41467-023-38901-y)
Supplement: Supplementary file 5 — Supplementary Data 2 [file 41467_2023_38901_MOESM5_ESM.zip › Supplementary Data 2_Animated images with detected wildebeest.pptx]

## Slide 1
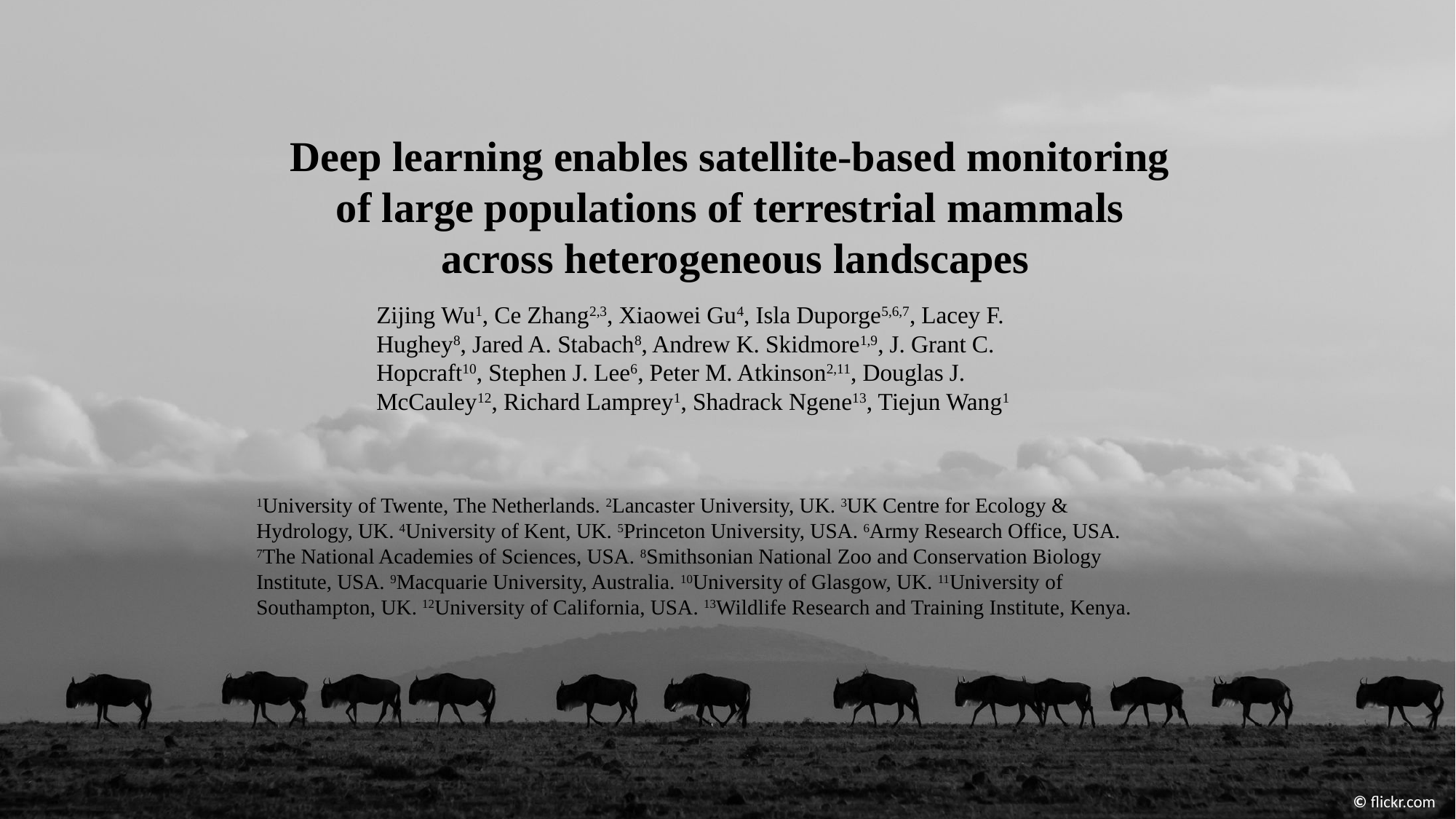

Deep learning enables satellite-based monitoring
of large populations of terrestrial mammals
across heterogeneous landscapes
Zijing Wu1, Ce Zhang2,3, Xiaowei Gu4, Isla Duporge5,6,7, Lacey F. Hughey8, Jared A. Stabach8, Andrew K. Skidmore1,9, J. Grant C. Hopcraft10, Stephen J. Lee6, Peter M. Atkinson2,11, Douglas J. McCauley12, Richard Lamprey1, Shadrack Ngene13, Tiejun Wang1
1University of Twente, The Netherlands. 2Lancaster University, UK. 3UK Centre for Ecology & Hydrology, UK. 4University of Kent, UK. 5Princeton University, USA. 6Army Research Office, USA. 7The National Academies of Sciences, USA. 8Smithsonian National Zoo and Conservation Biology Institute, USA. 9Macquarie University, Australia. 10University of Glasgow, UK. 11University of Southampton, UK. 12University of California, USA. 13Wildlife Research and Training Institute, Kenya.
© flickr.com

## Slide 2
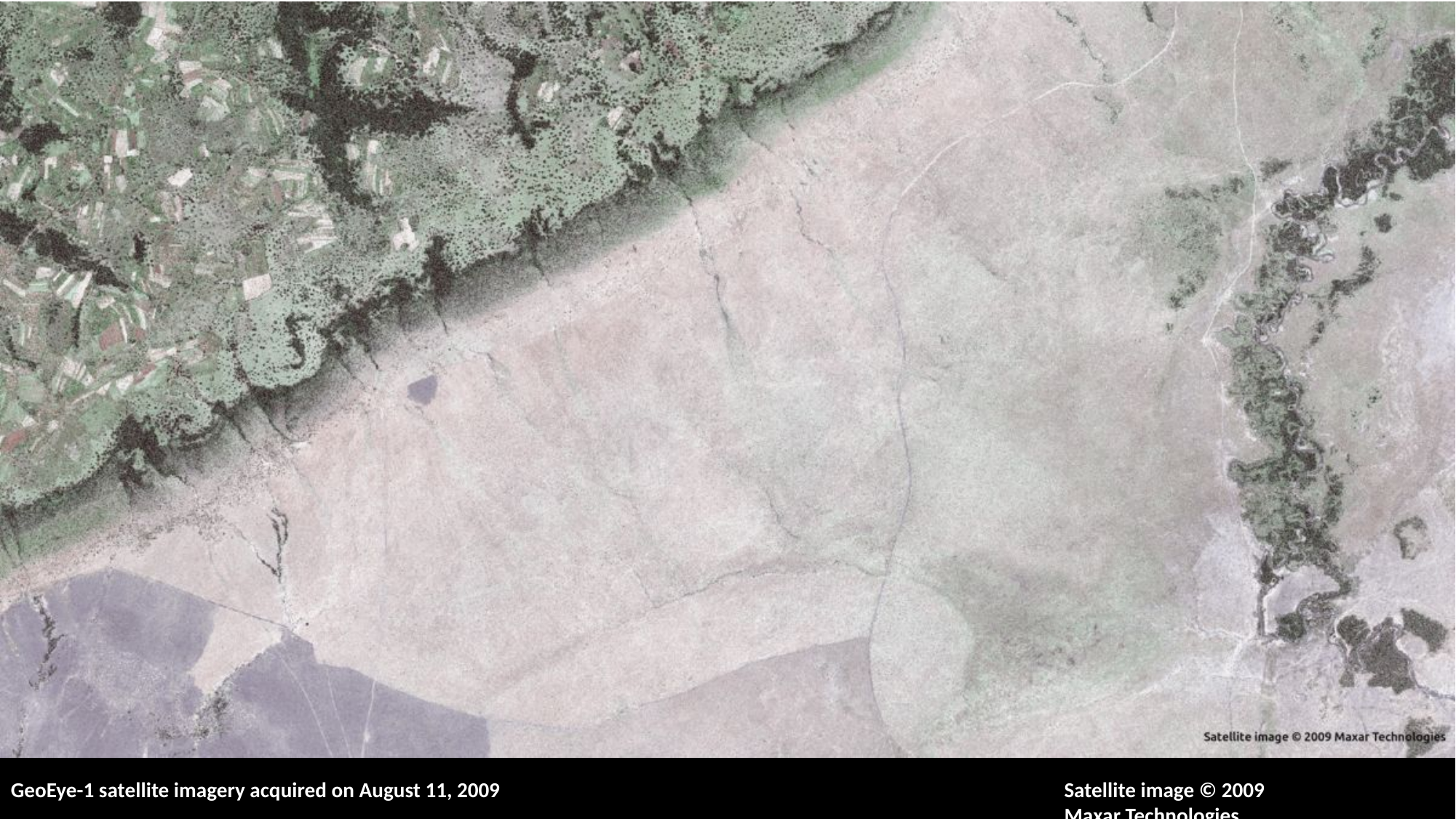

GeoEye-1 satellite imagery acquired on August 11, 2009
Satellite image © 2009 Maxar Technologies

## Slide 3
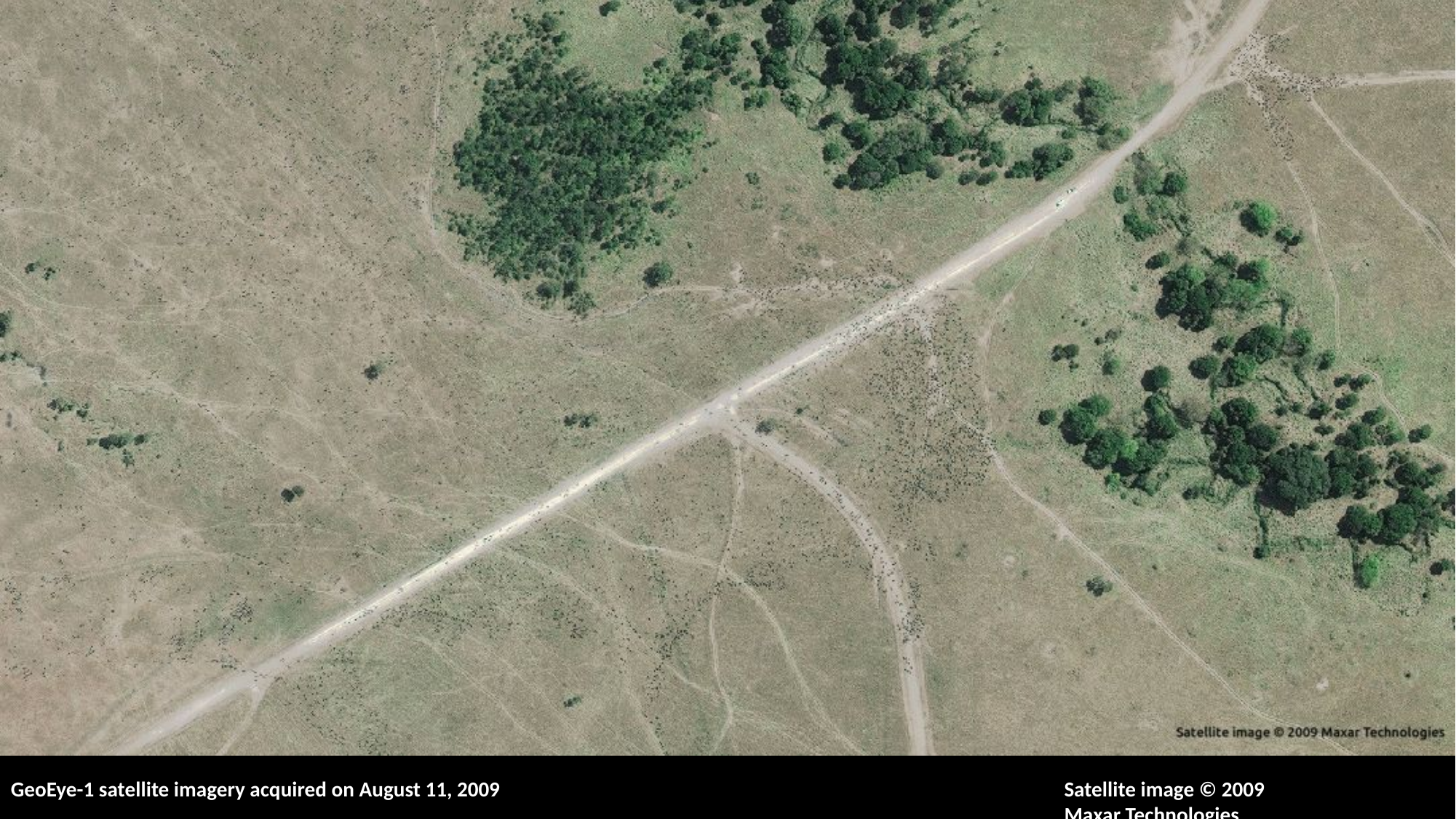

GeoEye-1 satellite imagery acquired on August 11, 2009
Satellite image © 2009 Maxar Technologies

## Slide 4
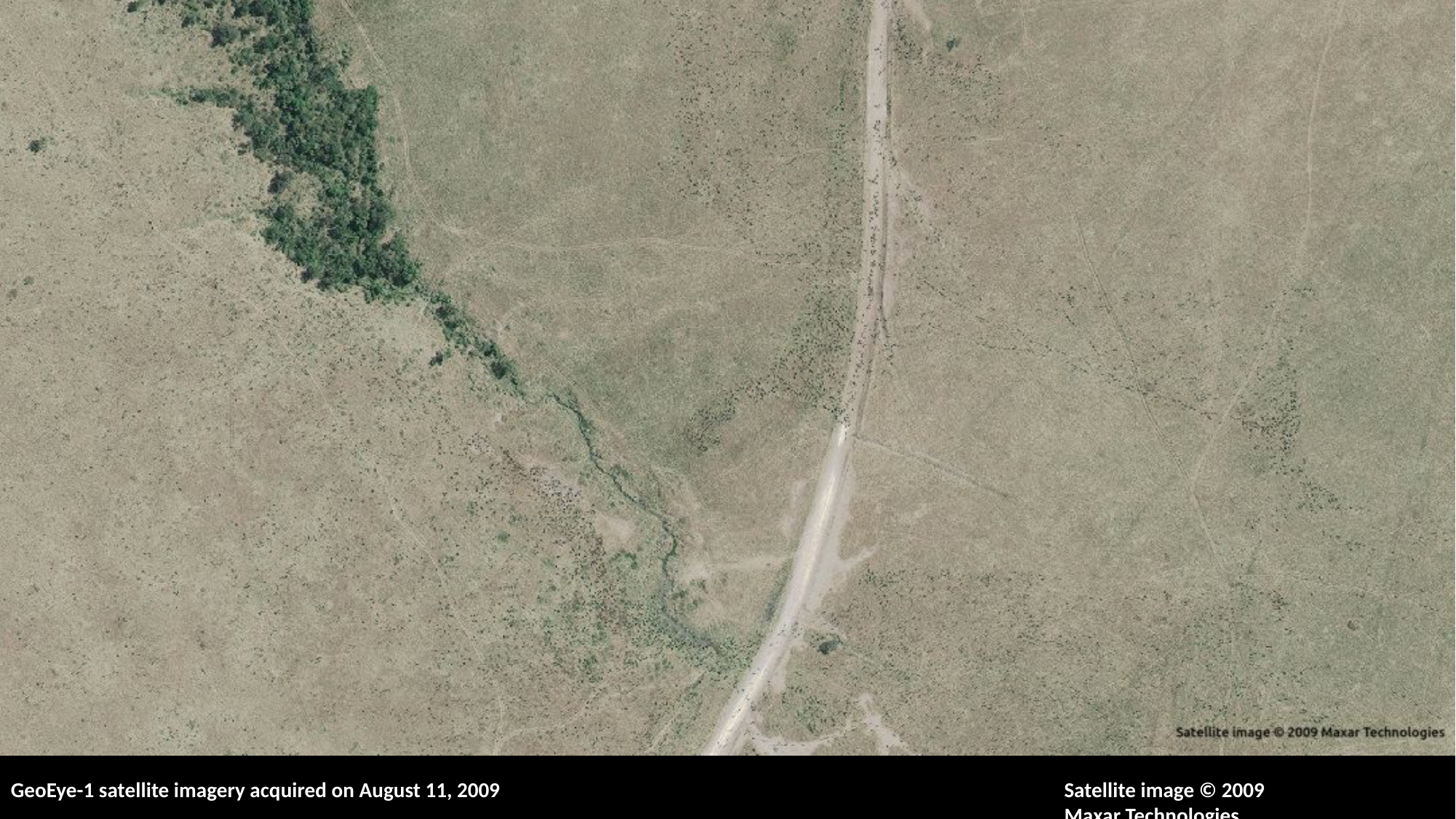

GeoEye-1 satellite imagery acquired on August 11, 2009
Satellite image © 2009 Maxar Technologies

## Slide 5
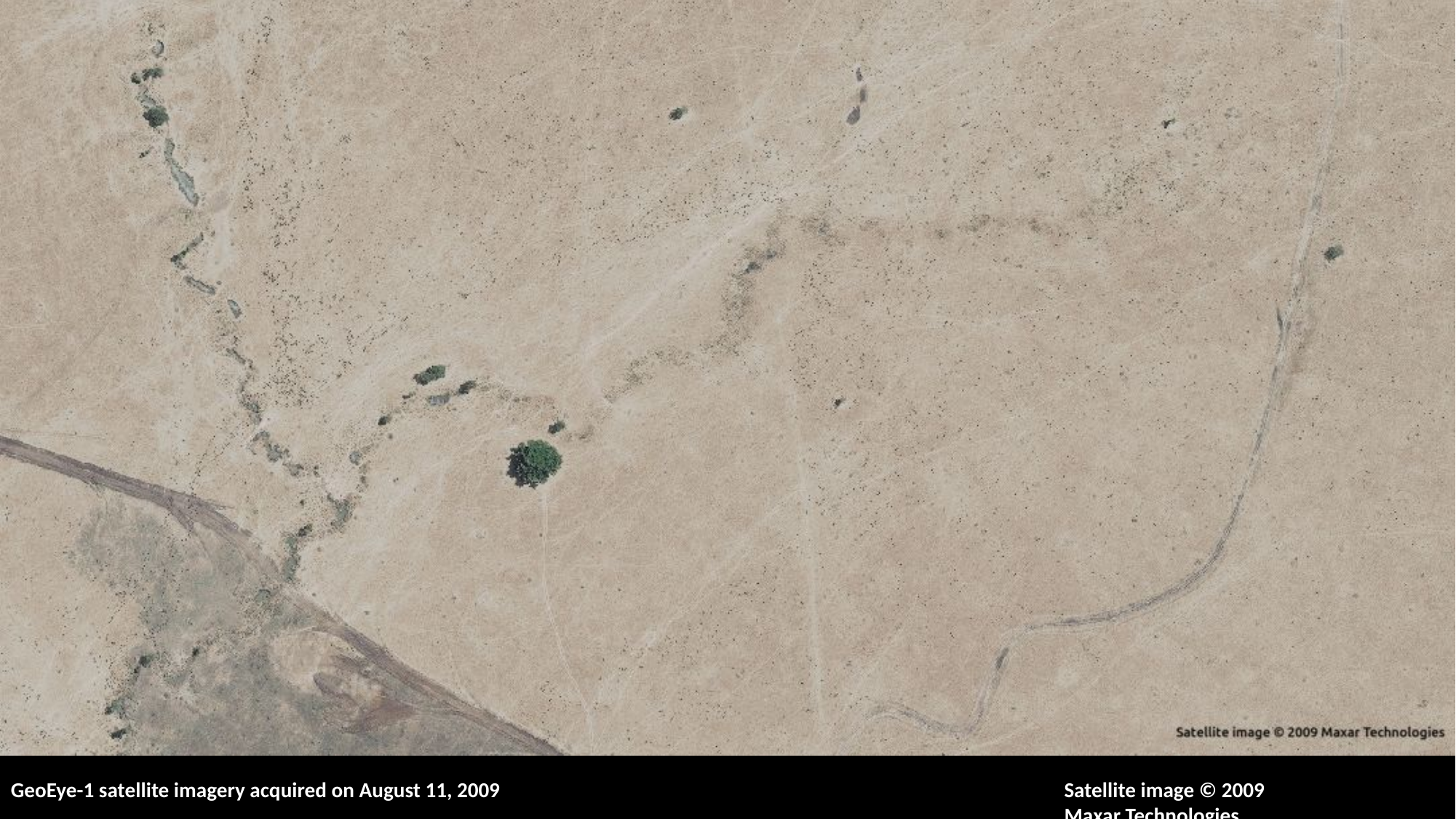

GeoEye-1 satellite imagery acquired on August 11, 2009
Satellite image © 2009 Maxar Technologies

## Slide 6
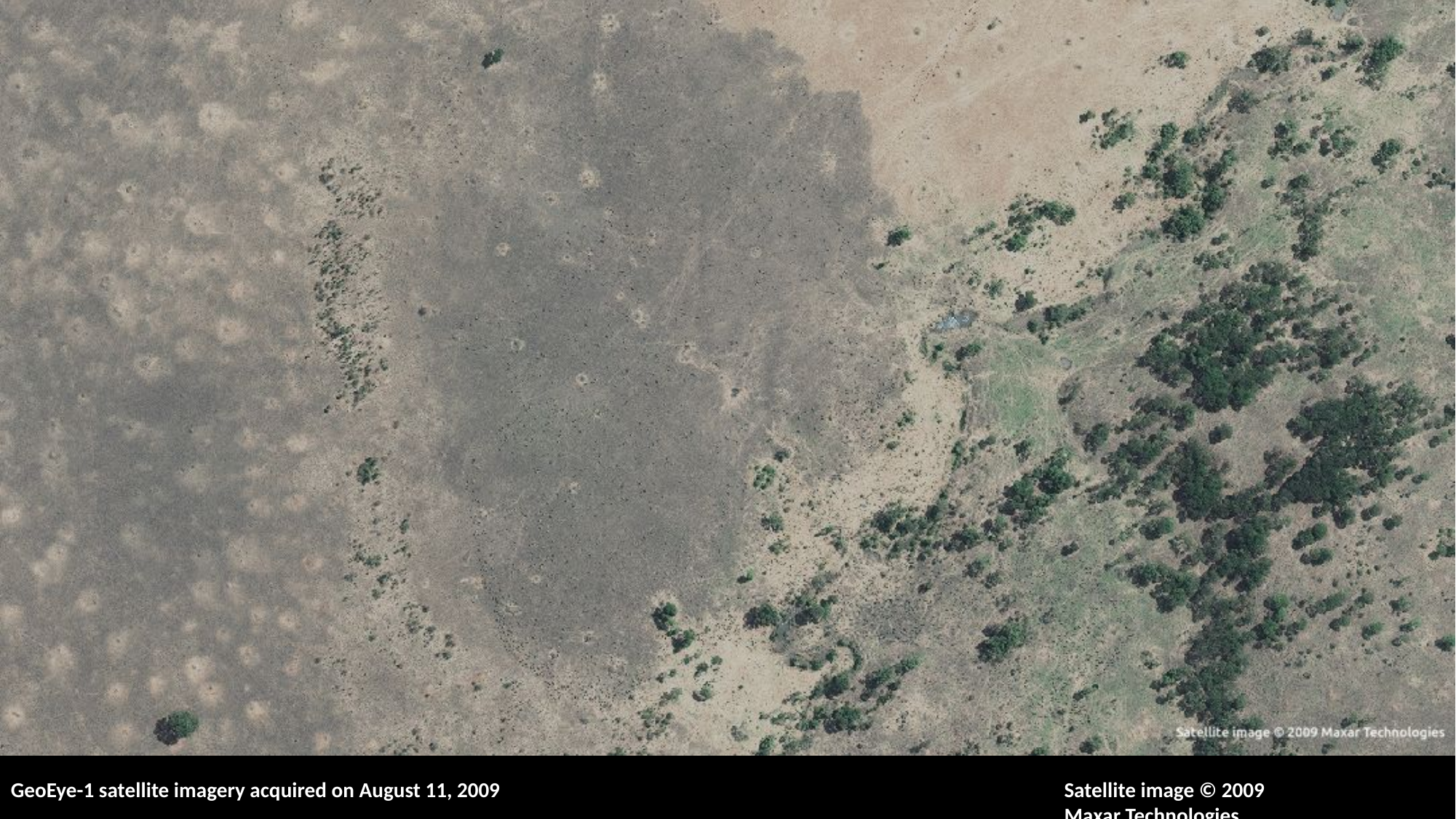

GeoEye-1 satellite imagery acquired on August 11, 2009
Satellite image © 2009 Maxar Technologies

## Slide 7
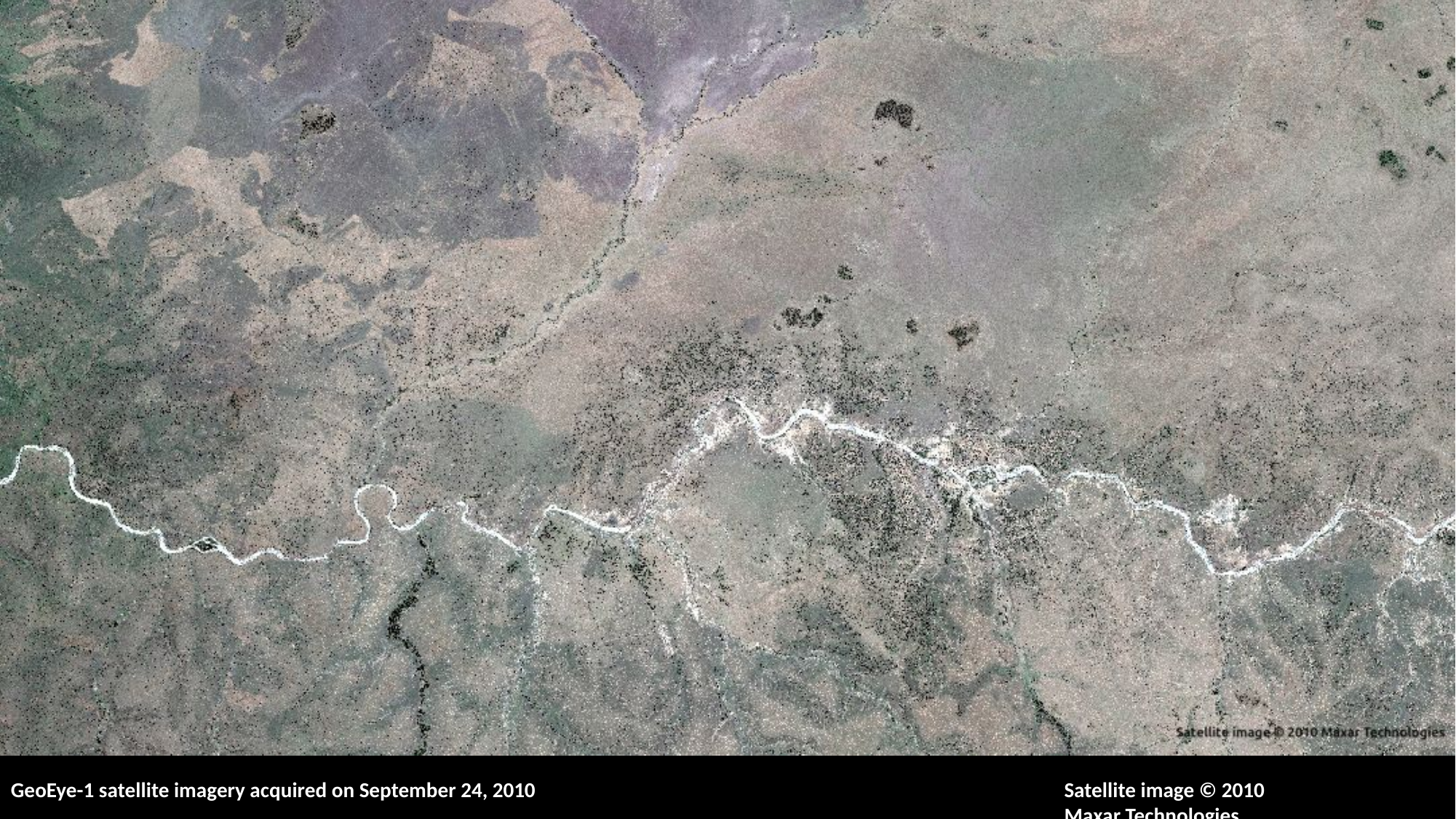

GeoEye-1 satellite imagery acquired on September 24, 2010
Satellite image © 2010 Maxar Technologies

## Slide 8
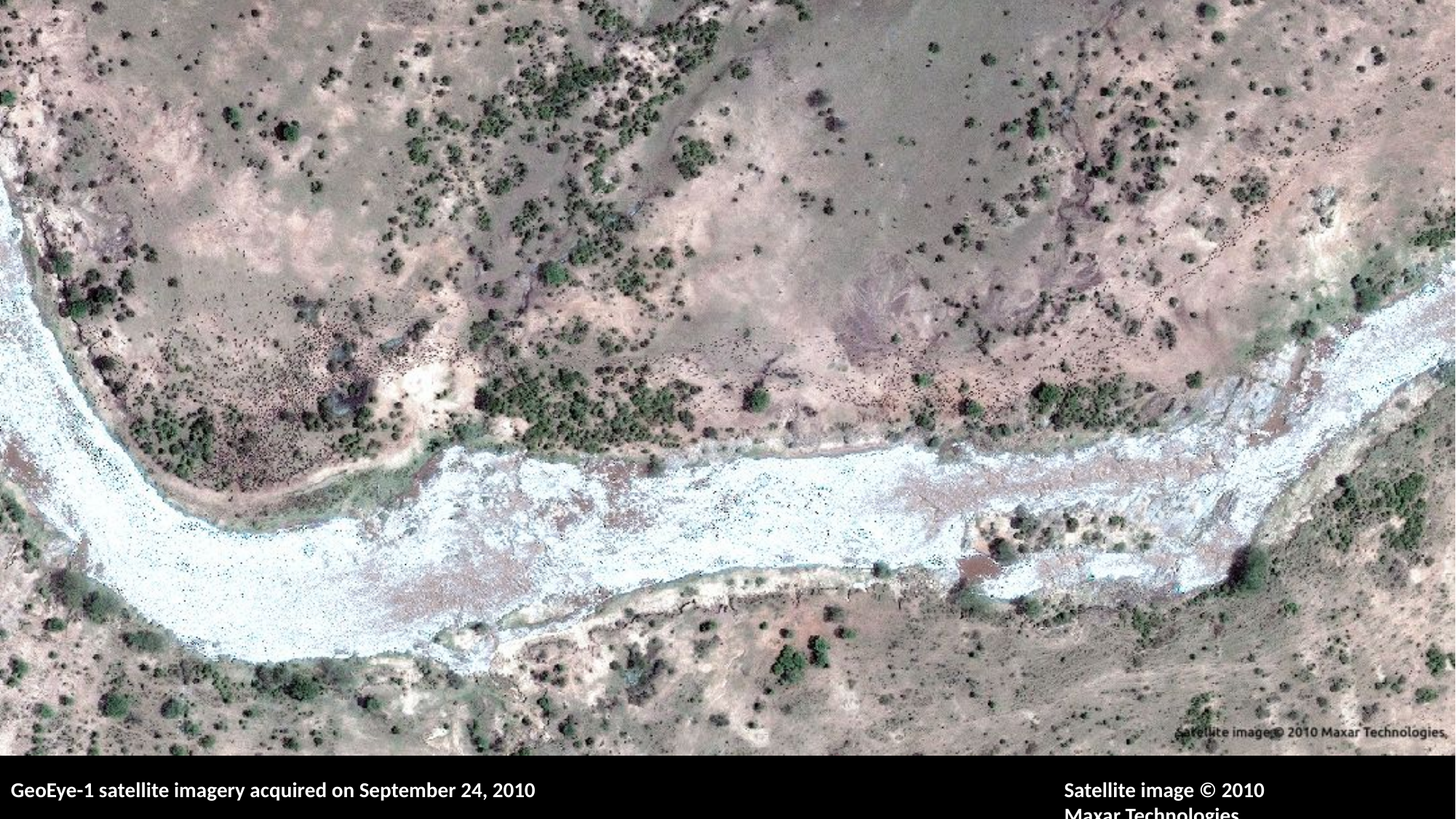

GeoEye-1 satellite imagery acquired on September 24, 2010
Satellite image © 2010 Maxar Technologies

## Slide 9
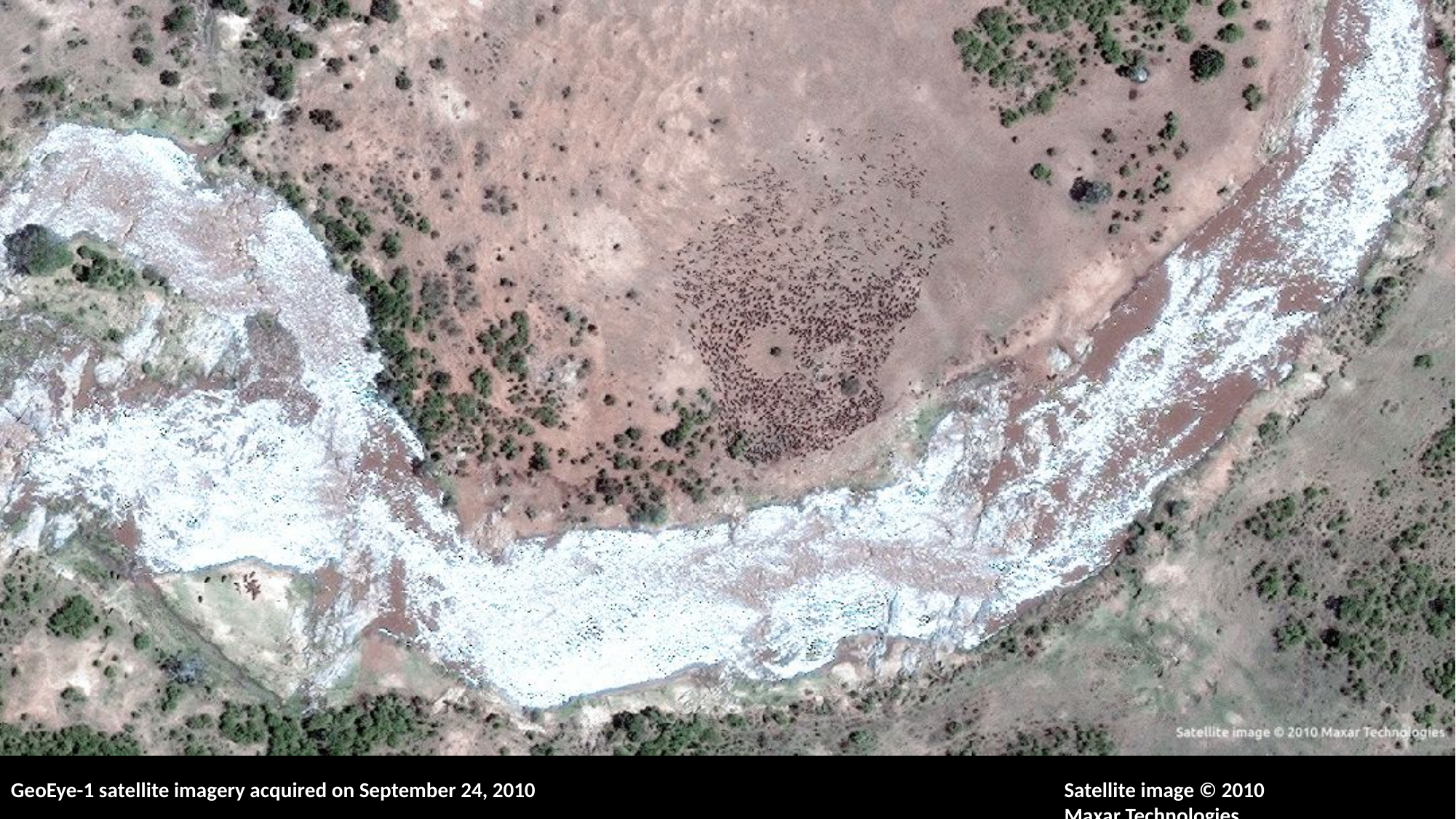

GeoEye-1 satellite imagery acquired on September 24, 2010
Satellite image © 2010 Maxar Technologies

## Slide 10
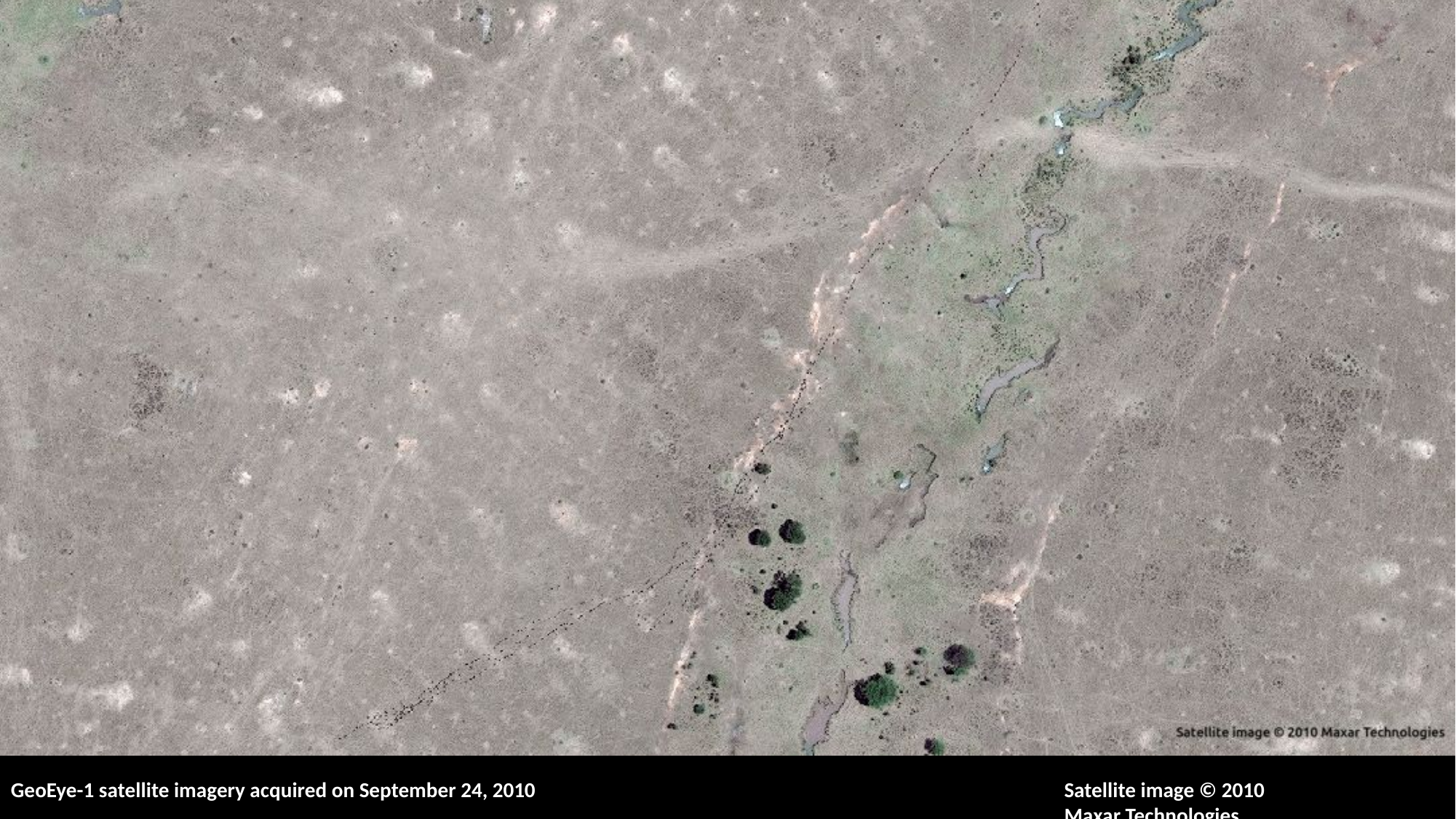

GeoEye-1 satellite imagery acquired on September 24, 2010
Satellite image © 2010 Maxar Technologies

## Slide 11
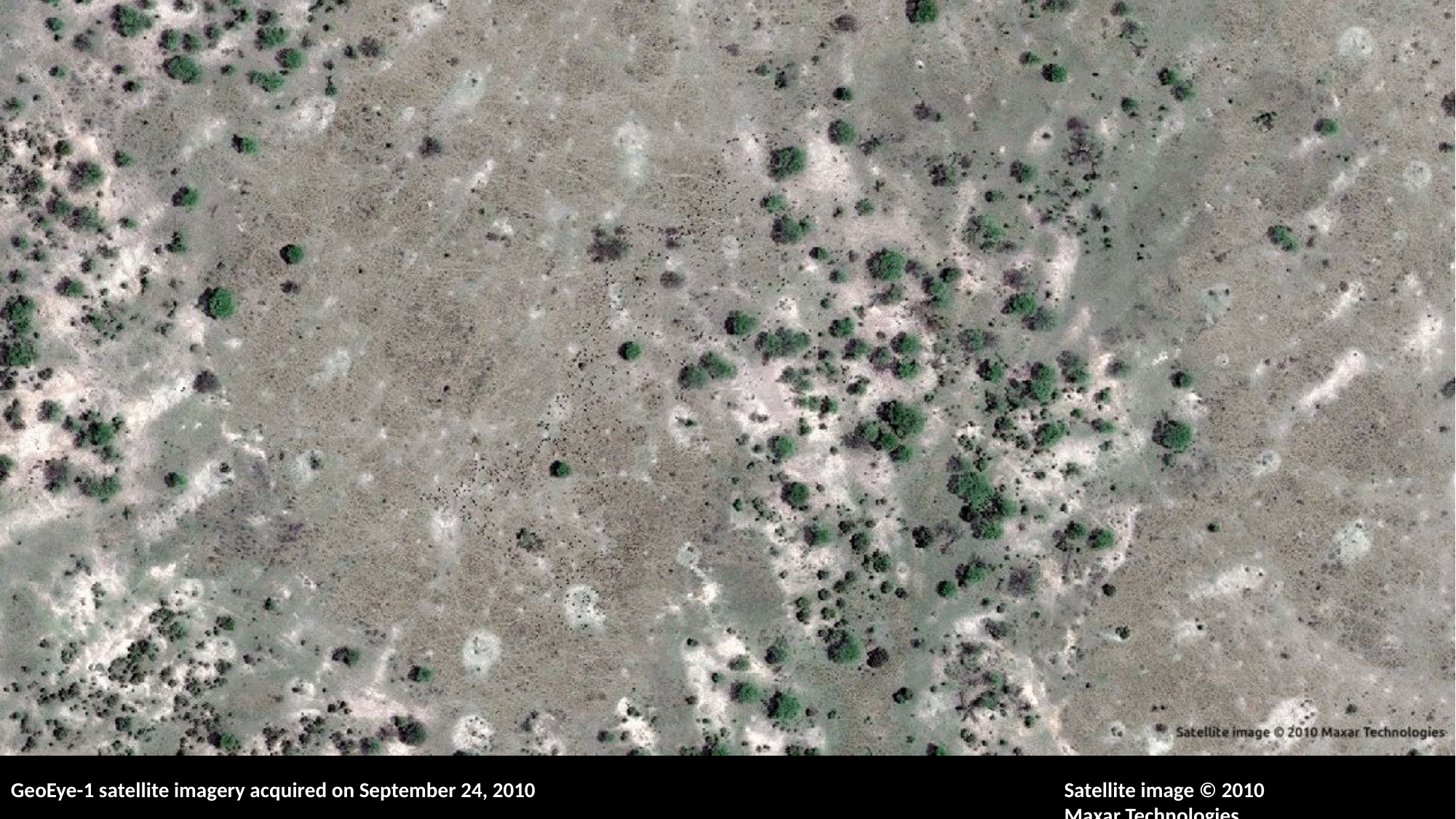

GeoEye-1 satellite imagery acquired on September 24, 2010
Satellite image © 2010 Maxar Technologies

## Slide 12
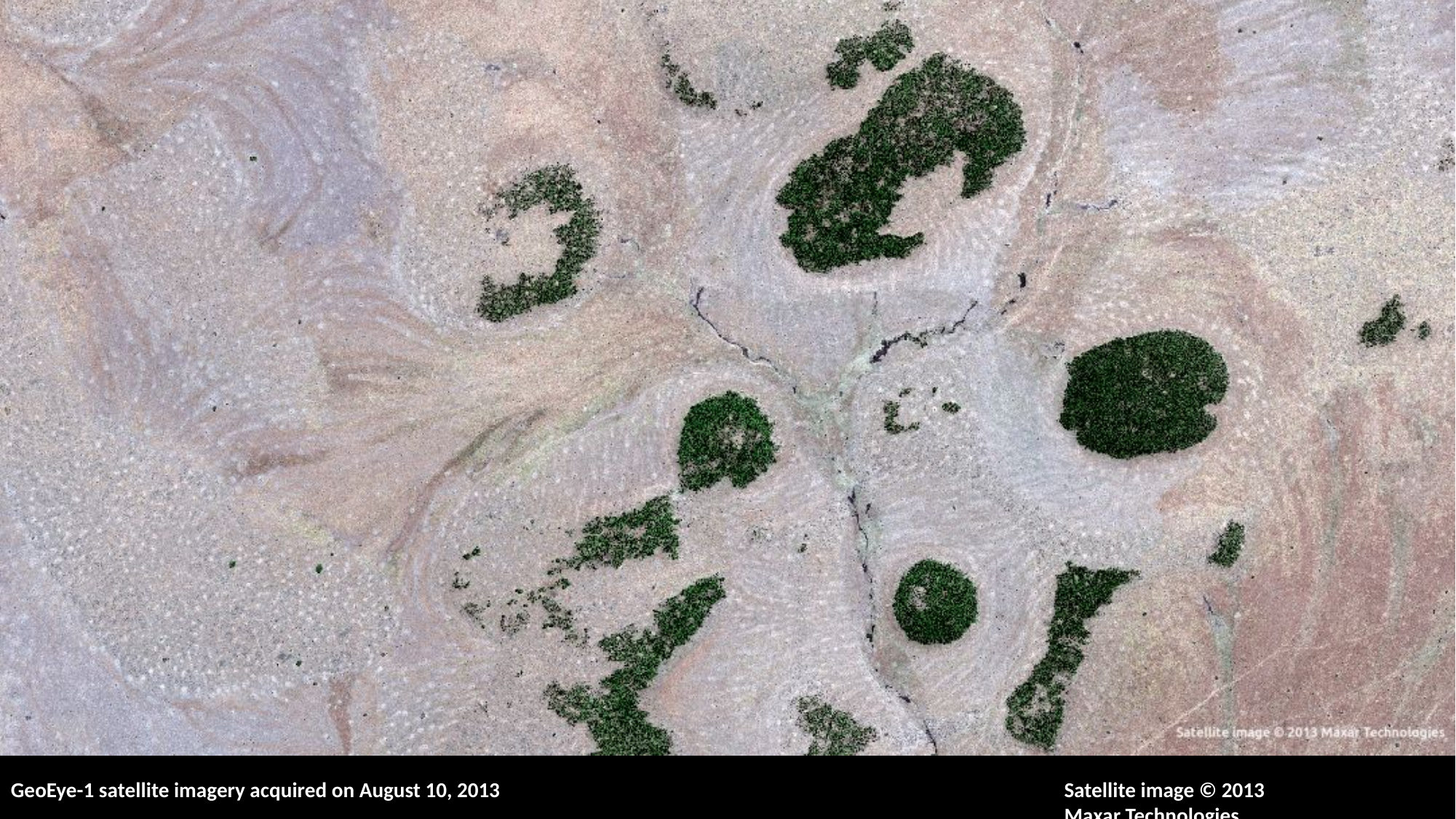

GeoEye-1 satellite imagery acquired on August 10, 2013
Satellite image © 2013 Maxar Technologies

## Slide 13
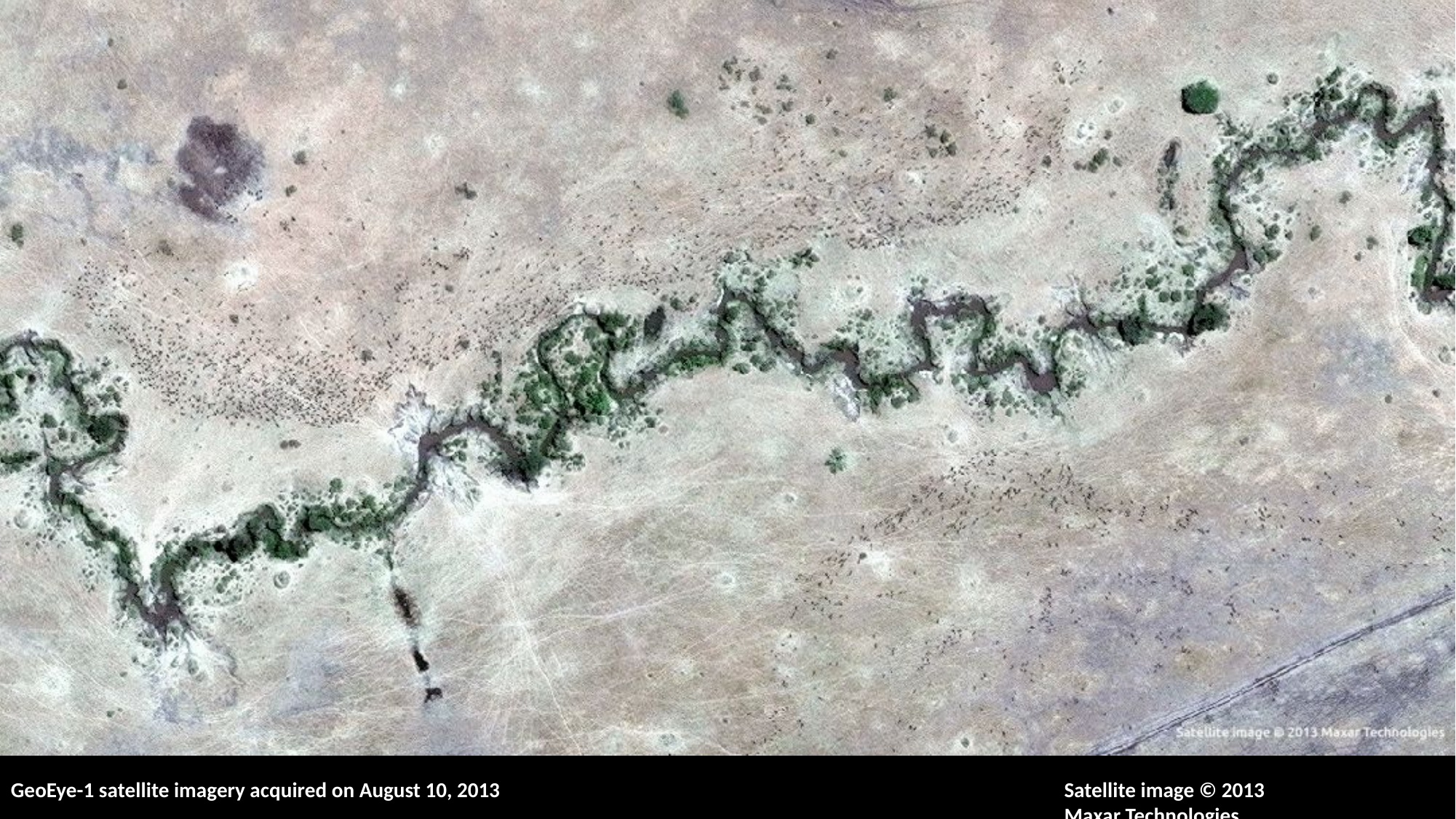

GeoEye-1 satellite imagery acquired on August 10, 2013
Satellite image © 2013 Maxar Technologies

## Slide 14
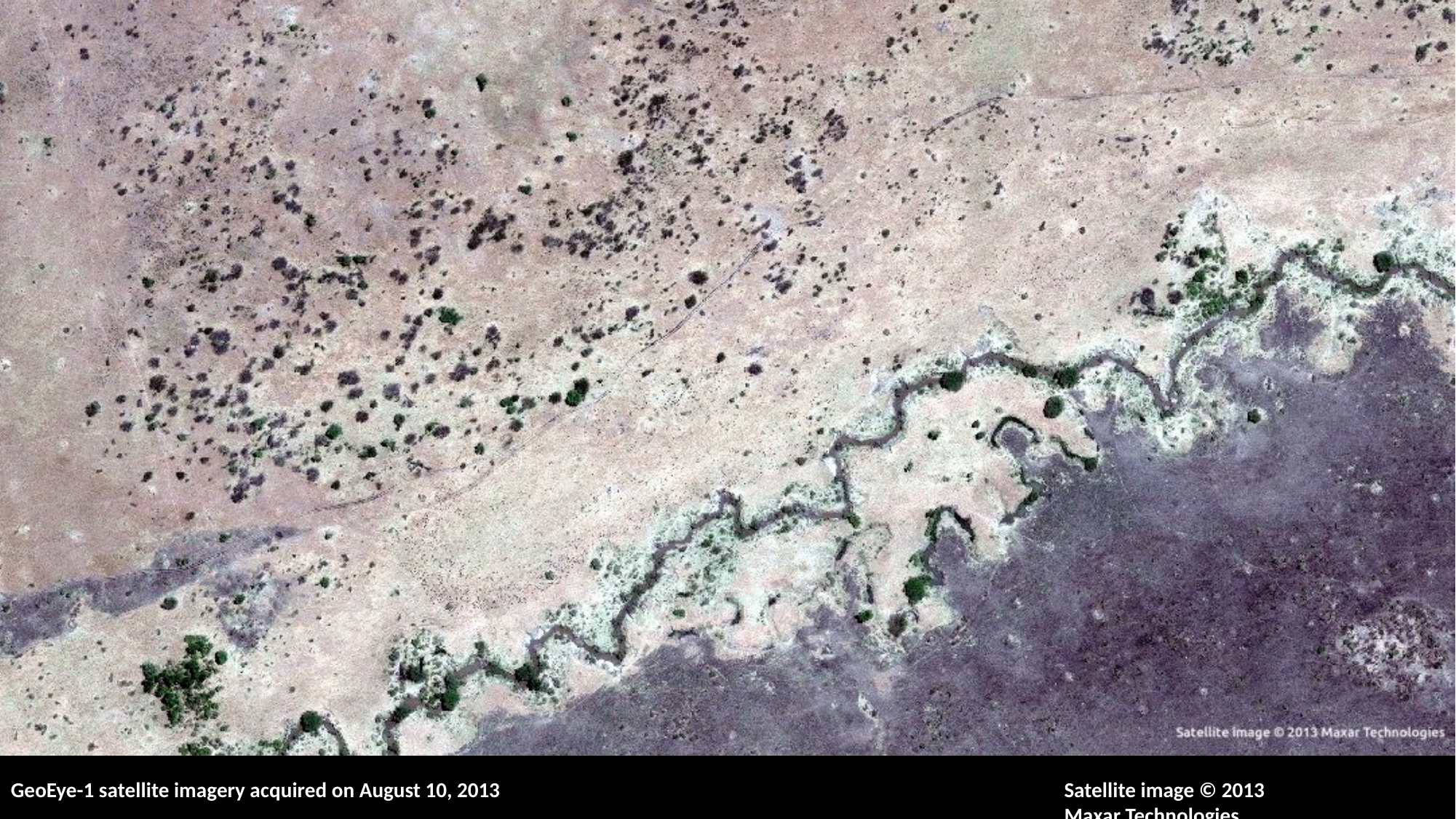

GeoEye-1 satellite imagery acquired on August 10, 2013
Satellite image © 2013 Maxar Technologies

## Slide 15
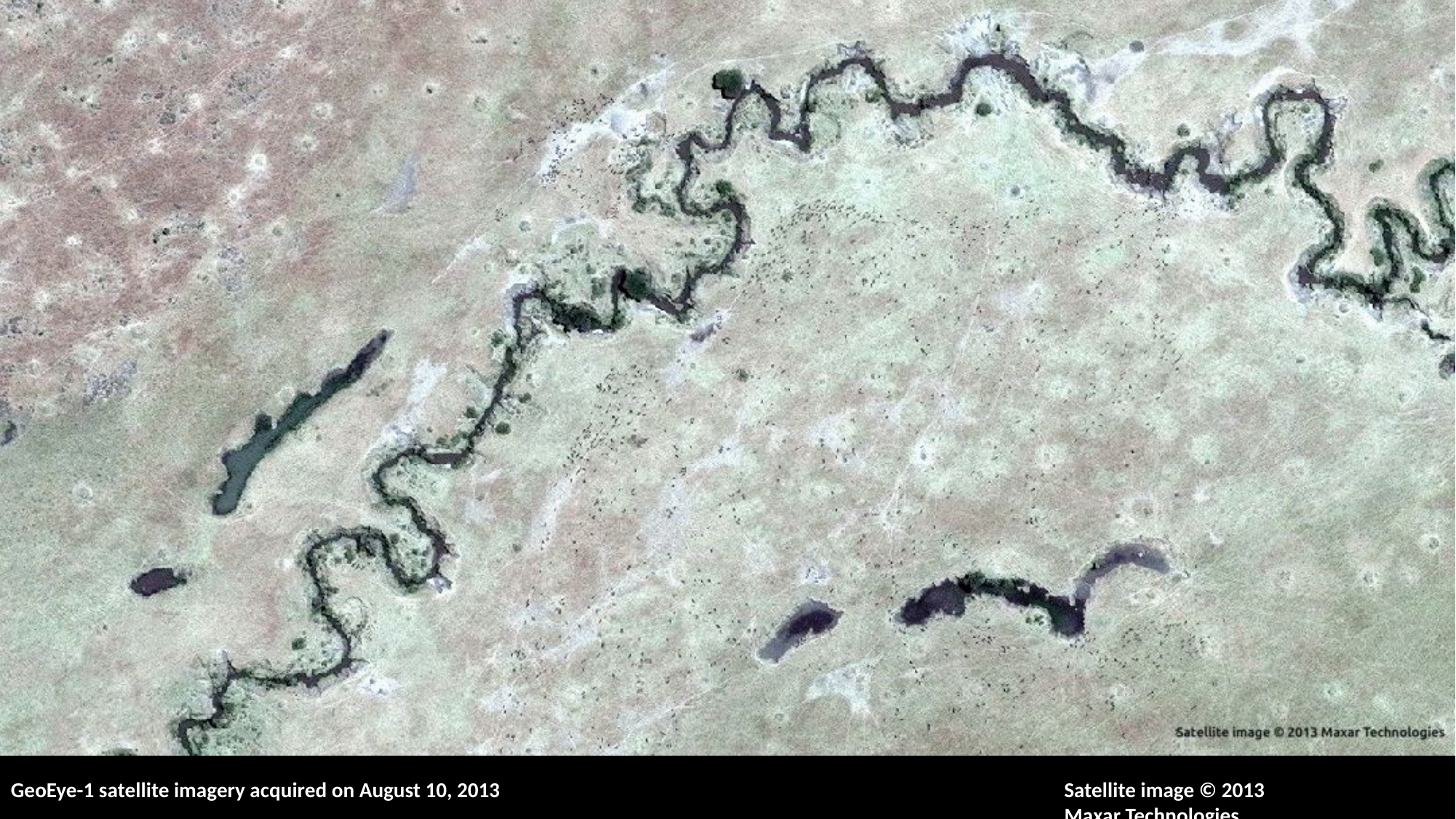

GeoEye-1 satellite imagery acquired on August 10, 2013
Satellite image © 2013 Maxar Technologies

## Slide 16
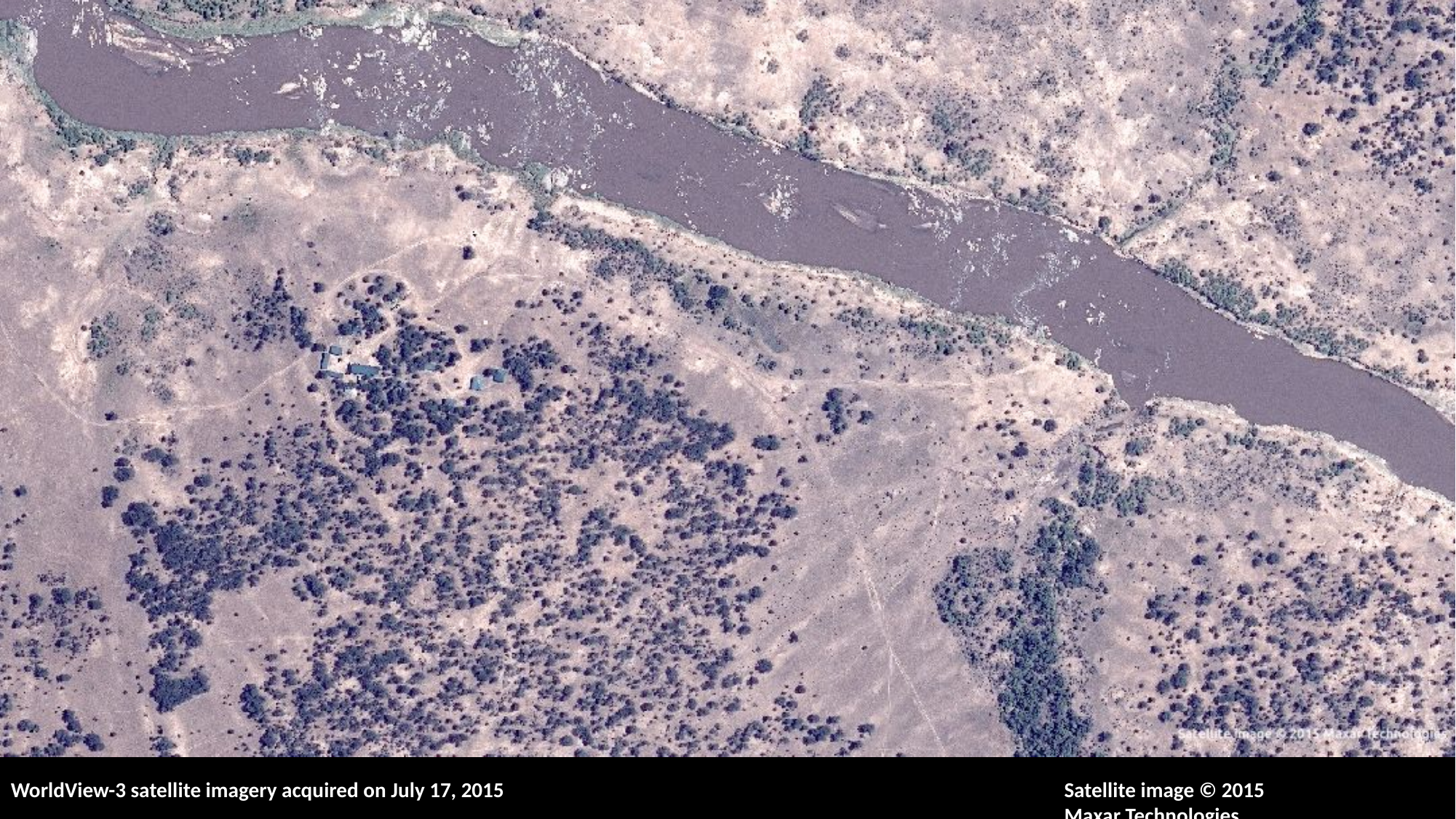

WorldView-3 satellite imagery acquired on July 17, 2015
Satellite image © 2015 Maxar Technologies

## Slide 17
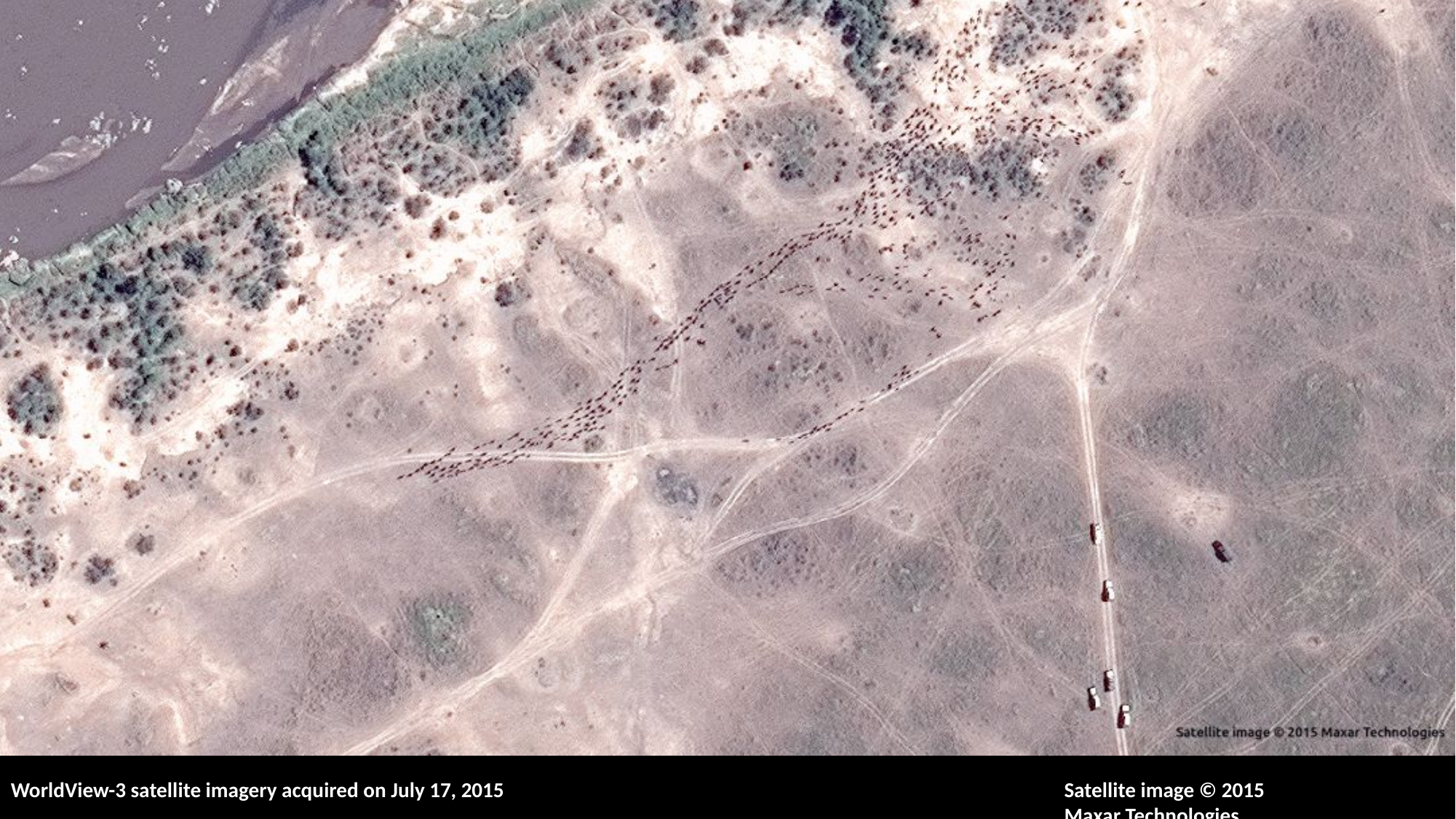

WorldView-3 satellite imagery acquired on July 17, 2015
Satellite image © 2015 Maxar Technologies

## Slide 18
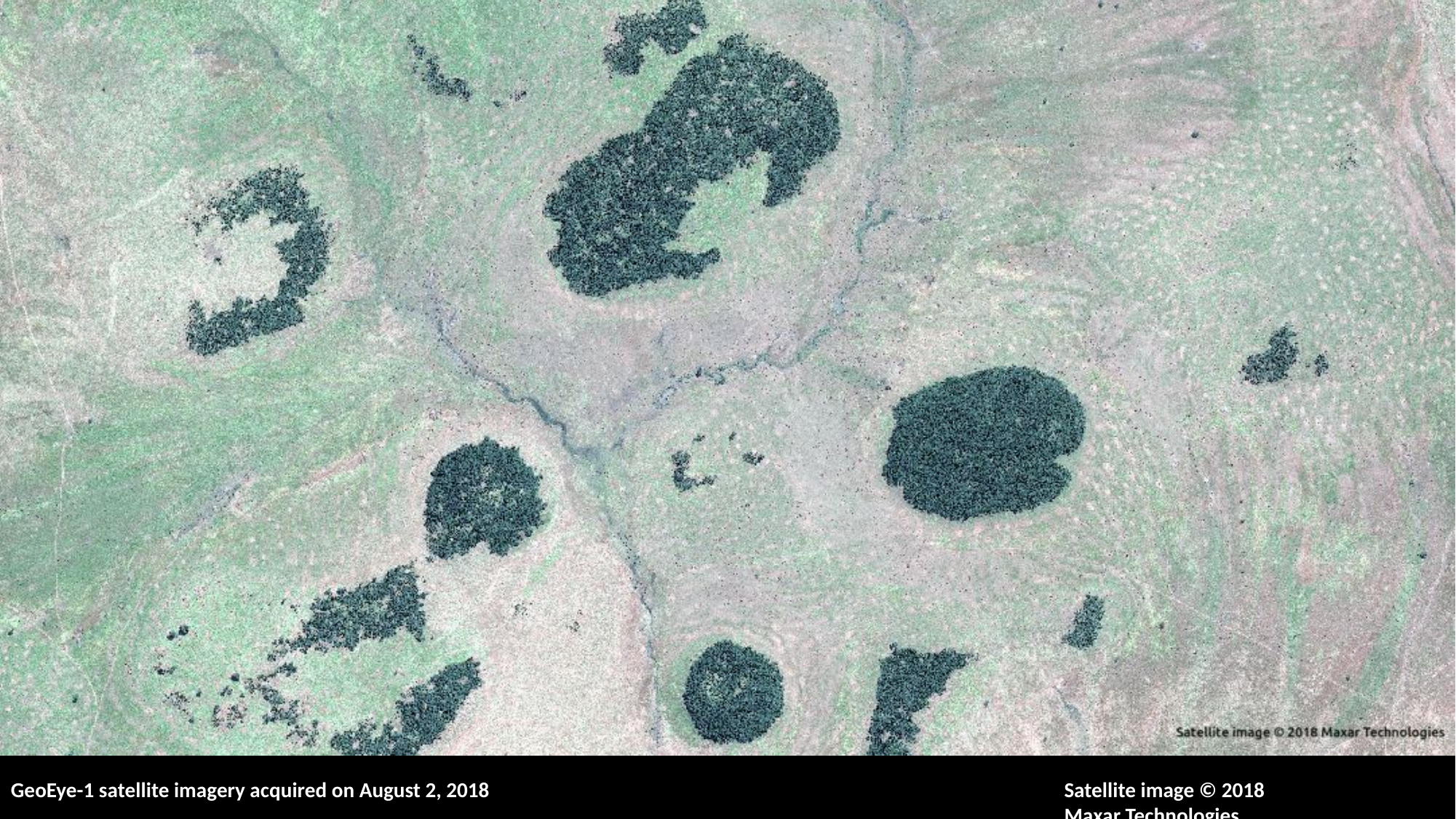

GeoEye-1 satellite imagery acquired on August 2, 2018
Satellite image © 2018 Maxar Technologies

## Slide 19
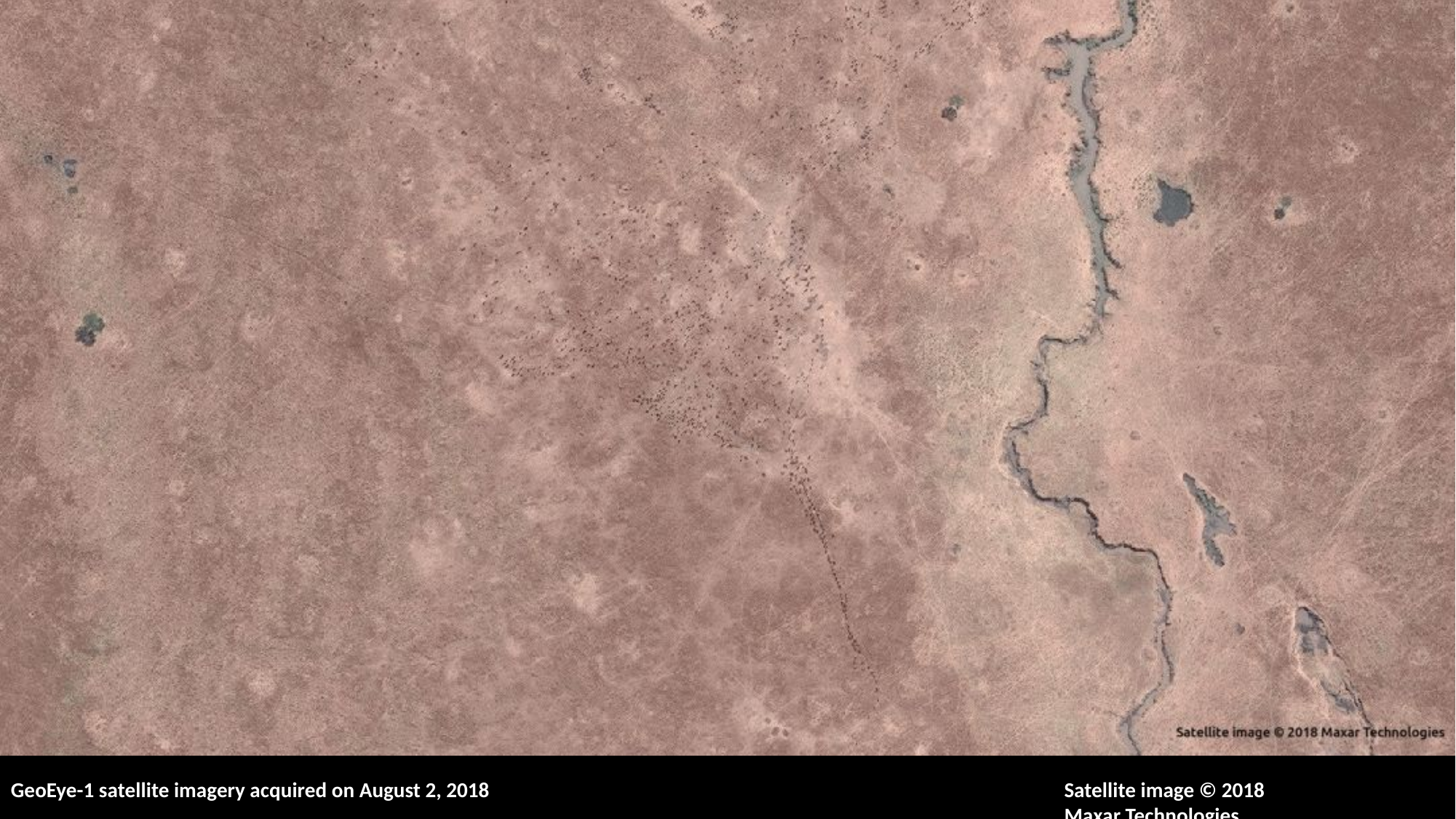

GeoEye-1 satellite imagery acquired on August 2, 2018
Satellite image © 2018 Maxar Technologies

## Slide 20
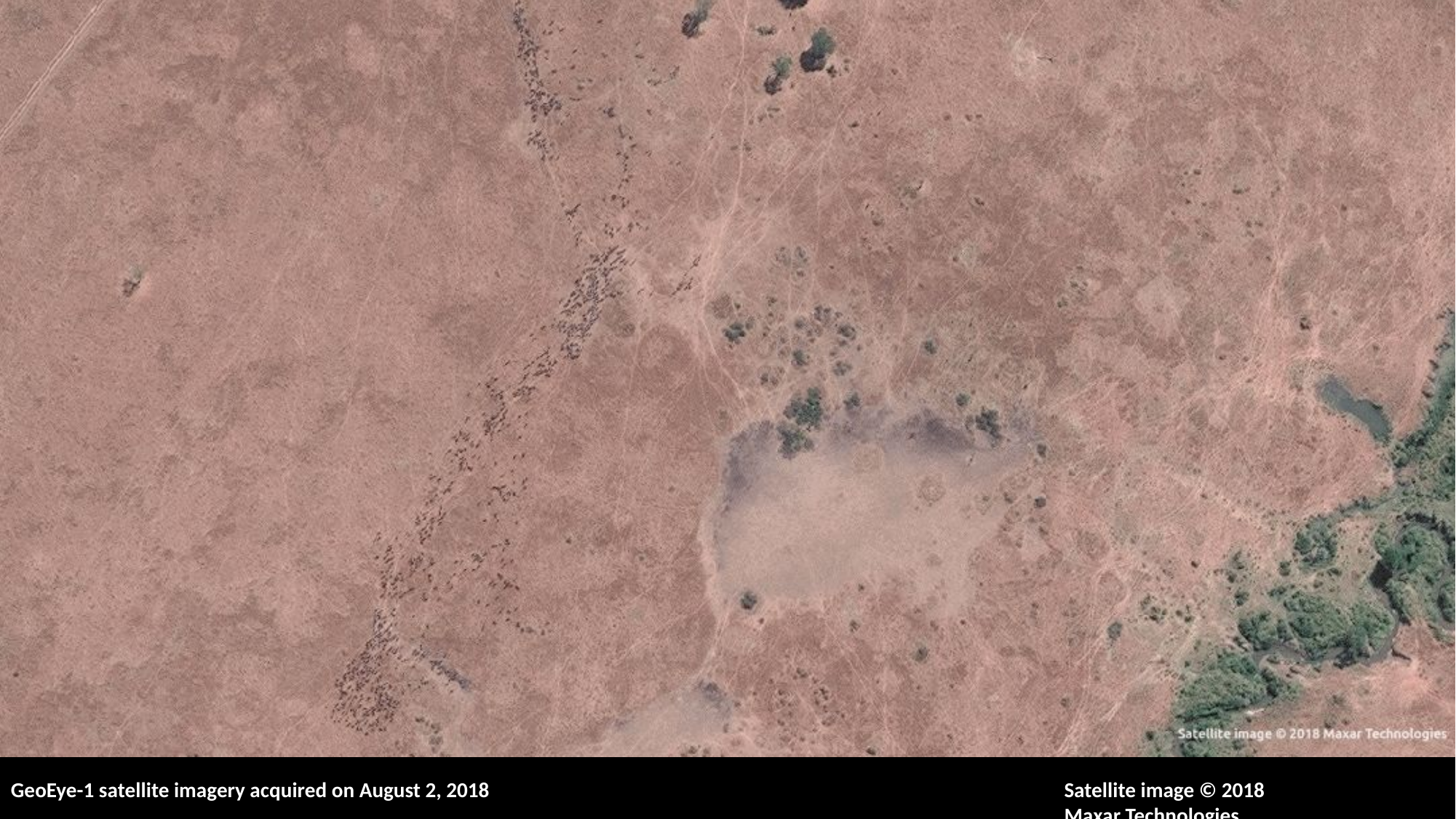

GeoEye-1 satellite imagery acquired on August 2, 2018
Satellite image © 2018 Maxar Technologies

## Slide 21
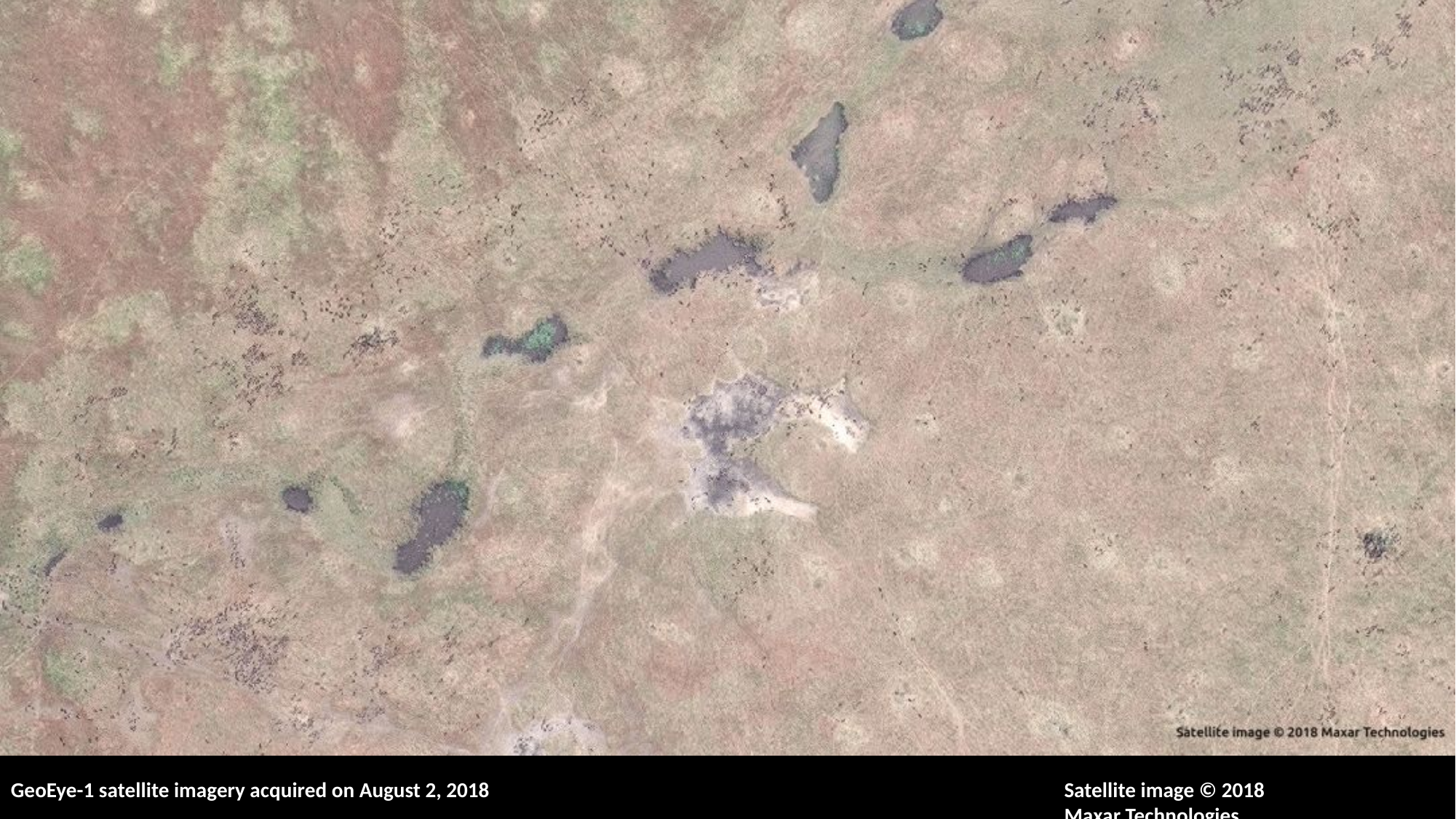

GeoEye-1 satellite imagery acquired on August 2, 2018
Satellite image © 2018 Maxar Technologies

## Slide 22
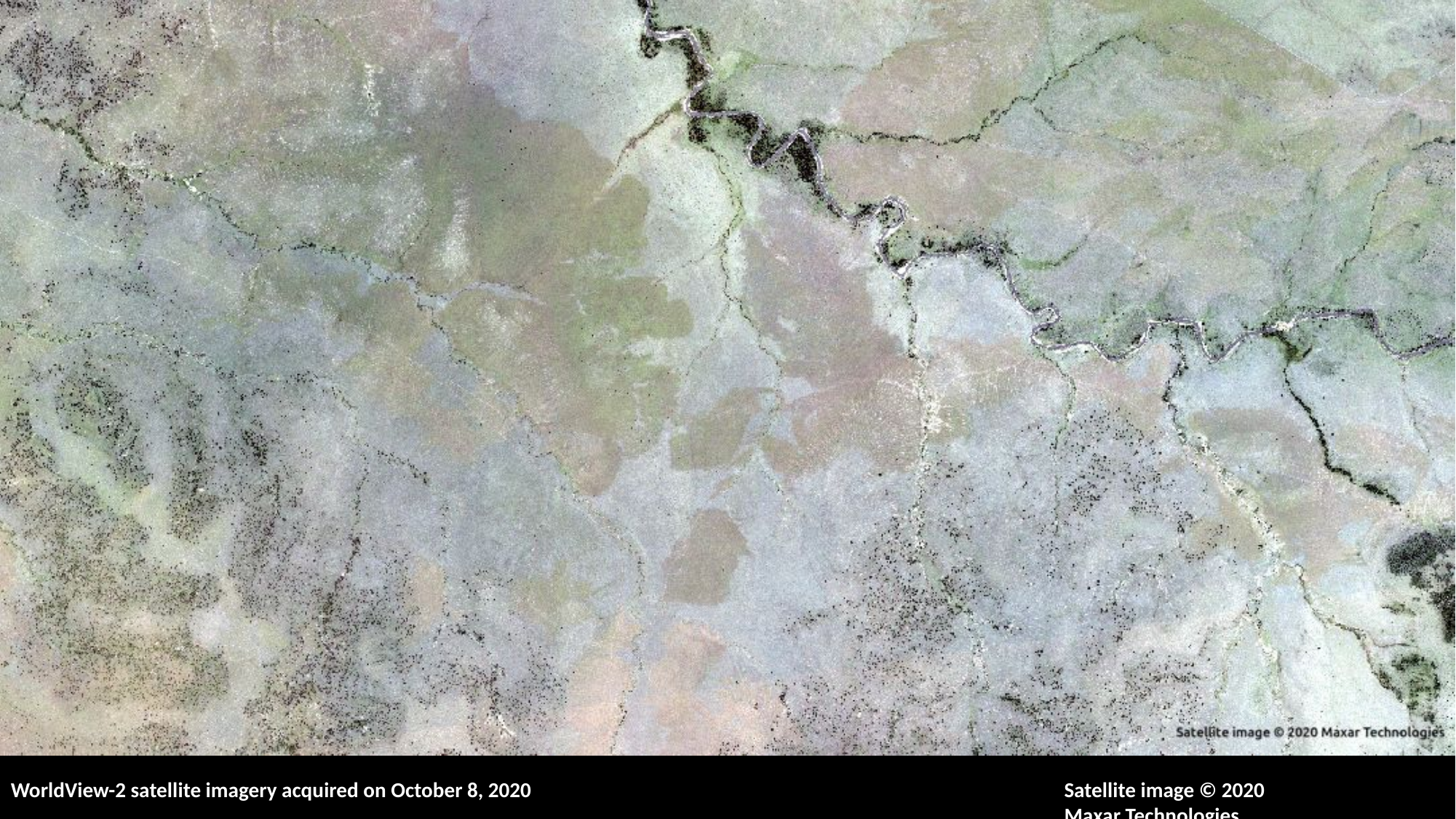

WorldView-2 satellite imagery acquired on October 8, 2020
Satellite image © 2020 Maxar Technologies

## Slide 23
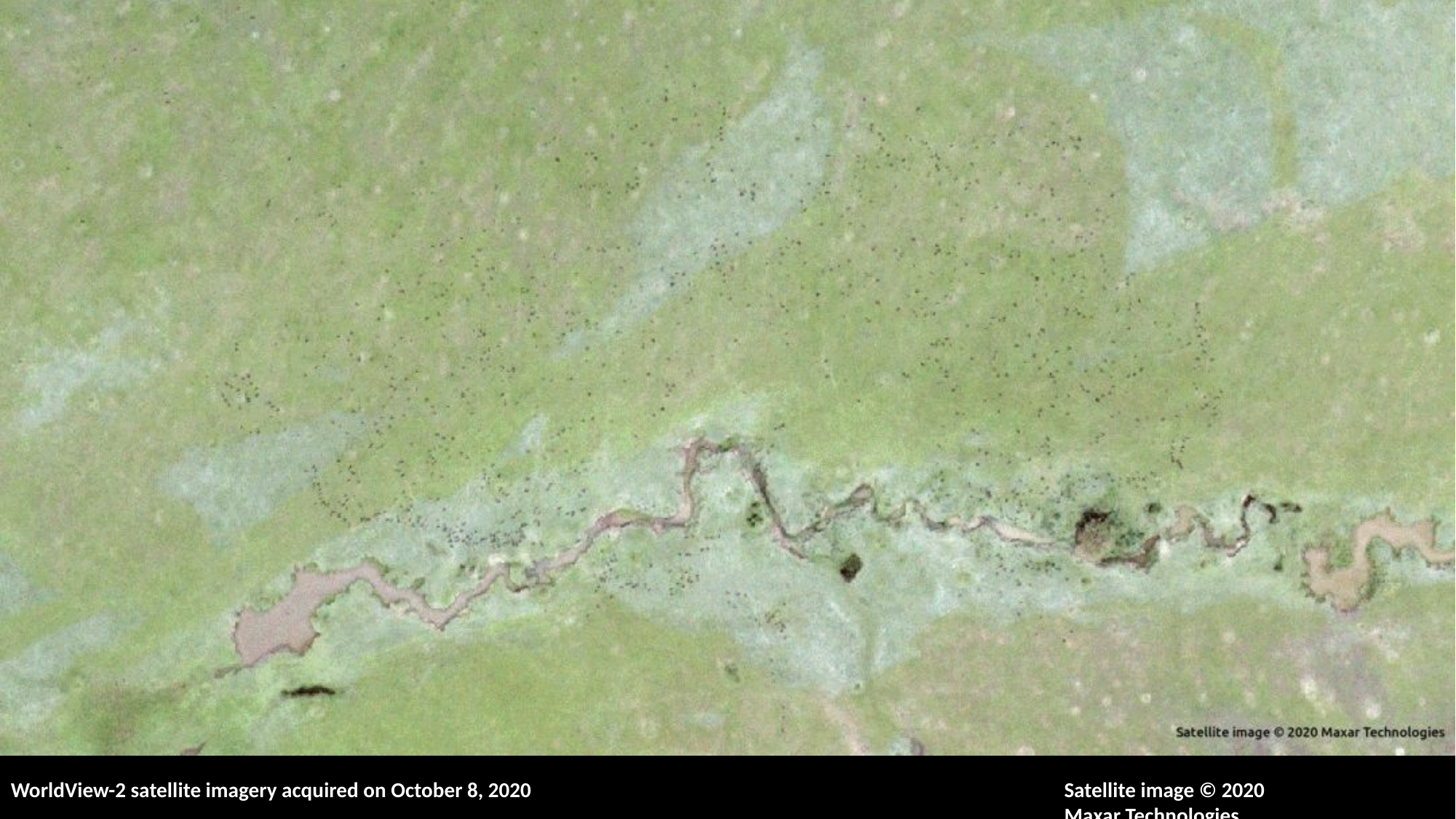

WorldView-2 satellite imagery acquired on October 8, 2020
Satellite image © 2020 Maxar Technologies

## Slide 24
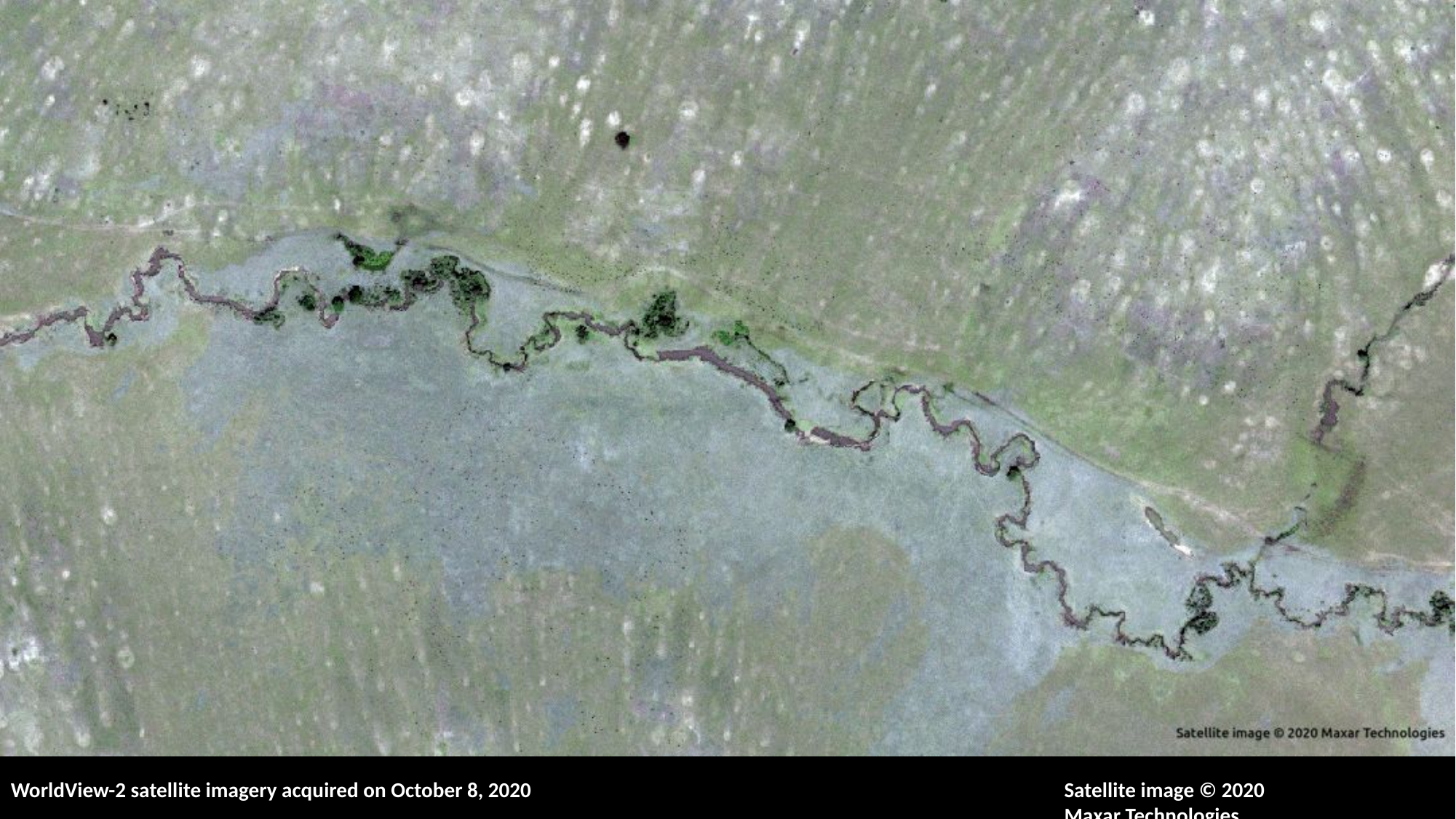

WorldView-2 satellite imagery acquired on October 8, 2020
Satellite image © 2020 Maxar Technologies

## Slide 25
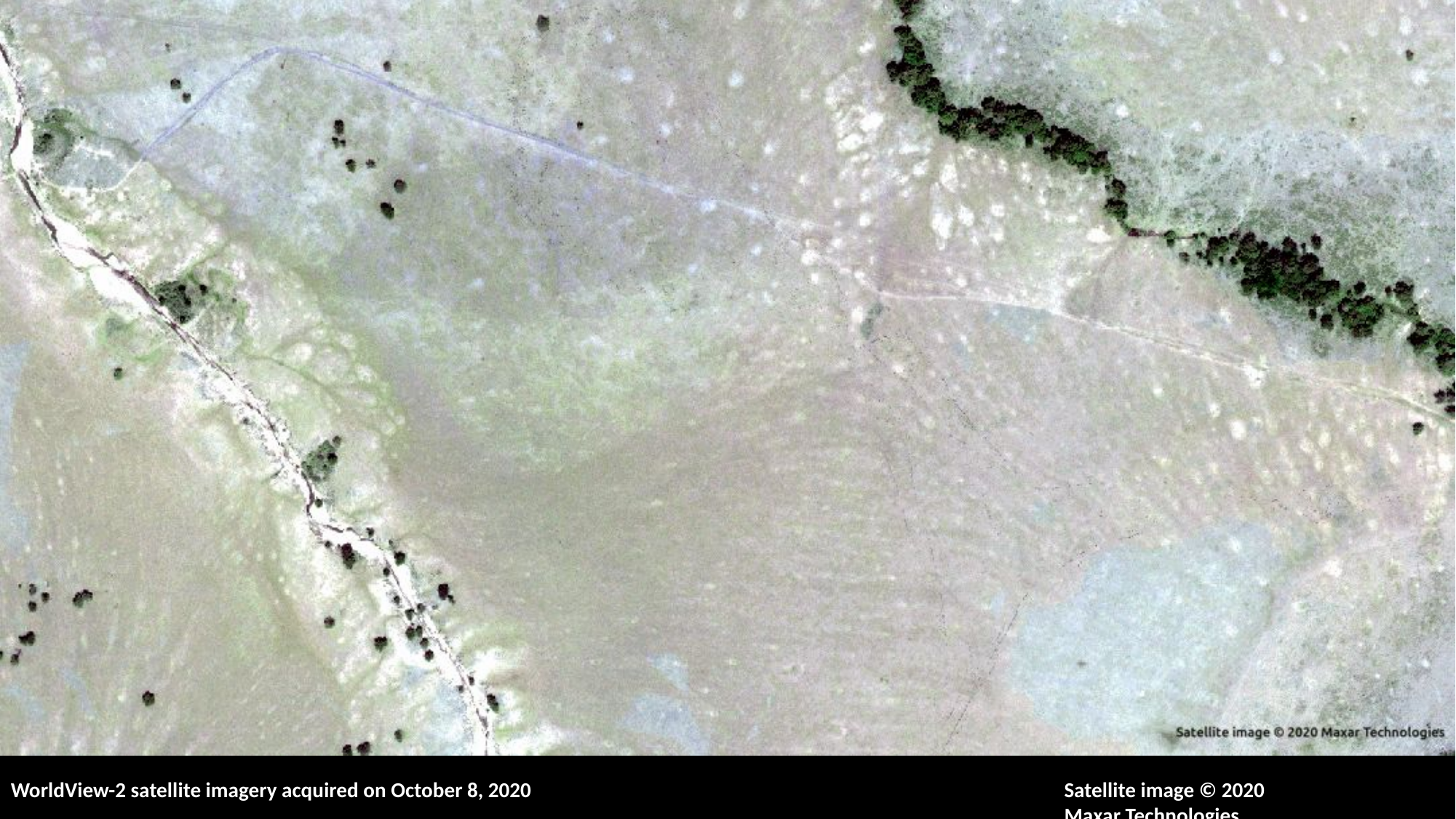

WorldView-2 satellite imagery acquired on October 8, 2020
Satellite image © 2020 Maxar Technologies

## Slide 26
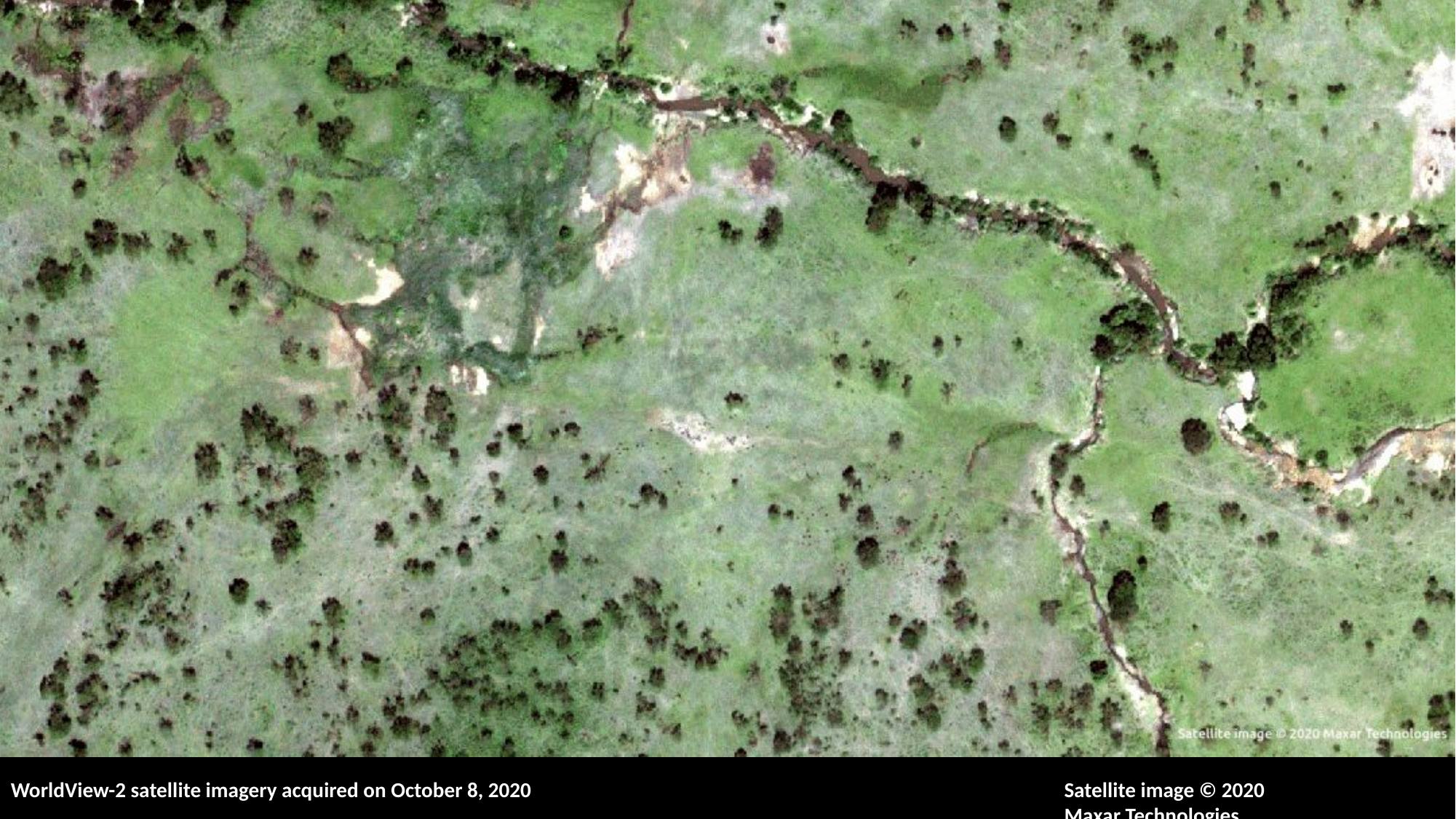

WorldView-2 satellite imagery acquired on October 8, 2020
Satellite image © 2020 Maxar Technologies
