# Supplementary material for: Deep learning enables satellite-based monitoring of large populations of terrestrial mammals across heterogeneous landscape
Source: Nat Commun. 2023 May 27;14:3072. doi: 10.1038/s41467-023-38901-y (PMC10224963; doi:10.1038/s41467-023-38901-y)
Supplement: Supplementary file 6 — Reporting Summary [file 41467_2023_38901_MOESM6_ESM.pdf]

nature portfolio

Corresponding author(s): Tiejun Wang
Last updated by author(s): Apr 5, 2023

Reporting Summary

Nature Portfolio wishes to improve the reproducibility of the work that we publish. This form provides structure for consistency and transparency in reporting. For further information on Nature Portfolio policies, see our Editorial Policies and the Editorial Policy Checklist.

Statistics

For all statistical analyses, confirm that the following items are present in the figure legend, table legend, main text, or Methods section.

n/a	Confirmed	
☐	☒	The exact sample size (*n*) for each experimental group/condition, given as a discrete number and unit of measurement
☐	☒	A statement on whether measurements were taken from distinct samples or whether the same sample was measured repeatedly
☒	☐	The statistical test(s) used AND whether they are one- or two-sided<br>*Only common tests should be described solely by name; describe more complex techniques in the Methods section.*
☒	☐	A description of all covariates tested
☒	☐	A description of any assumptions or corrections, such as tests of normality and adjustment for multiple comparisons
☐	☒	A full description of the statistical parameters including central tendency (e.g. means) or other basic estimates (e.g. regression coefficient) AND variation (e.g. standard deviation) or associated estimates of uncertainty (e.g. confidence intervals)
☒	☐	For null hypothesis testing, the test statistic (e.g. *F*, *t*, *r*) with confidence intervals, effect sizes, degrees of freedom and *P* value noted<br>*Give P values as exact values whenever suitable.*
☒	☐	For Bayesian analysis, information on the choice of priors and Markov chain Monte Carlo settings
☒	☐	For hierarchical and complex designs, identification of the appropriate level for tests and full reporting of outcomes
☒	☐	Estimates of effect sizes (e.g. Cohen's *d*, Pearson's *r*), indicating how they were calculated

Our web collection on statistics for biologists contains articles on many of the points above.

Software and code

Policy information about availability of computer code

Data collection

No special software was used to collect the data.

Data analysis

PCI Geomatica commercial software was used for pansharpening the WorldView-2 satellite images in 2020. Commercial software ArcGIS 10.8.2 was used to to display the satellite images, prepare wildebeest annotations, design the sampling grids, select samples, crop the training and test image patches, analyze the hotspots and wildebeest distribution, and produce maps. Free and open source software/algorithms/ packages were used for model training, testing, and prediction: Python 3.7, GDAL tools 3.0.2, fiona 1.8.13, geopandas 0.8.1, imgaug 0.4.0, keras 2.4.3, numpy 1.16.5, opencv-python 4.5.3.56, pandas 1.1.5, rasterio 1.1.0, scikit-image 0.16.2, scikit-learn 0.23.2, shapely 1.7.1, The plots were produced using matplotlib 3.1.2 and seaborn 0.11.2. The deep learning code was written in Python (3.7.10) using Tensoflow (2.1.0) and ran on Google Colaboratory (for preliminary experiment) and Microsoft Azure platform using Anaconda. Custom algorithms were used to analyze the data and has been made available at Github (https://doi.org/10.5281/zenodo.7810487).

For manuscripts utilizing custom algorithms or software that are central to the research but not yet described in published literature, software must be made available to editors and reviewers. We strongly encourage code deposition in a community repository (e.g. GitHub). See the Nature Portfolio guidelines for submitting code & software for further information.

Data

Policy information about availability of data

All manuscripts must include a data availability statement. This statement should provide the following information, where applicable:

- Accession codes, unique identifiers, or web links for publicly available datasets
- A description of any restrictions on data availability
- For clinical datasets or third party data, please ensure that the statement adheres to our policy

All data needed to evaluate the conclusions in the paper are present in the paper and/or the Supplementary Materials. Source data are provided with this paper. The sample dataset that can be used to demonstrate the U-Net based wildebeest detection framework are publicly available at https://github.com/zijing-w/Wildebeest-UNet. Commercial very-fine-resolution satellite images were acquired by the Smithsonian Conservation Biology Institute and the United States Army Research Laboratory, under a NextView Imagery End User License Agreement. The copyright remains with Maxar Technologies (formally DigitalGlobe), and redistribution is not possible. Any further relevant data are available from the corresponding authors upon reasonable request.

Human research participants

Policy information about studies involving human research participants and Sex and Gender in Research.

Reporting on sex and gender	The analysis was conducted on wildlife detection using satellite imagery and there are no human research participants involved. Therefore, reporting on sex and gender is not relevant in this study.
Population characteristics	No human research participants are involved in this study.
Recruitment	No human research participants are involved in this study.
Ethics oversight	No human research participants are involved in this study.

Note that full information on the approval of the study protocol must also be provided in the manuscript.

Field-specific reporting

Please select the one below that is the best fit for your research. If you are not sure, read the appropriate sections before making your selection.

☐ Life sciences ☐ Behavioural & social sciences ☒ Ecological, evolutionary & environmental sciences

For a reference copy of the document with all sections, see nature.com/documents/nr-reporting-summary-flat.pdf

Ecological, evolutionary & environmental sciences study design

All studies must disclose on these points even when the disclosure is negative.

Study description	This study presents a framework for efficiently locating and counting wildebeest-sized animals with a body length of 1.5-2.5 m from submeter-resolution satellite imagery across a large, highly heterogeneous landscape. We developed a U-Net deep learning-based pipeline embedded with a post-processing clustering module, which uses high-precision pixel-based image segmentation to locate animals at the object level. We demonstrate the power of this framework by deploying it to satellite images covering a total area of 2,747 km2 across the Serengeti-Mara ecosystem and detected approximately 500,000 wildebeest individuals.
Research sample	We applied the pipeline to satellite images acquired over six years (2009, 2010, 2013, 2015, 2018, and 2020) covering 2,747 km2 in the Serengeti-Mara ecosystem. For each satellite image, we built a grid system with a cell size ranging from 150 m to 170 m, dependent on image resolution. Each grid covered at least 336 × 336 pixels, which was the size of the image patch for model training. The training and test datasets were sampled based on the cell units of the grid. For model training, we selected a total of 1097 training grids, covering different types of landscapes and various wildebeest abundances across all six years. The training dataset contains 53,906 wildebeest, occupying 27.13 km2, which is 0.7% of the whole area. For model testing, we used a stratified proportionate random sampling method to select test sample plots on each image date, containing 2700 sample grids with 11,594 wildebeest. The strata are based on the number of animals in the image patches. We adopted this method to guarantee the test dataset represent satellite images with very limited number of wildebeest as well as large number of wildebeest and varied landscapes. To detect and count migratory wildebeest within the area, we applied the model to the entire satellite imagery dataset to detect all the migratory wildebeest covered.
Sampling strategy	We used a stratified random sampling method to select test sample plots across the images, including 2700 test images containing 11,594 wildebeest individuals. The strata are based on the number of animals in the image patches. The samples contain image patches with different levels of animal density (from 0 to ~800 animals on 336 by 336 pixels) and thus the sample size is sufficient to represent the model performance.
Data collection	The satellite imagery used for wildebeest detection and counting includes nine multispectral images captured by three satellite

Data collection	sensors (GeoEye-1, WorldView-2 and WorldView-3) over six years in the Serengeti-Mara ecosystem. We selected these images from the archived very-fine-resolution satellite images acquired by the Maxar satellite constellation.
Timing and spatial scale	The satellite images were acquired over six years (August 2009, September 2010, August 2013, July 2015, August 2018, and October 2020) and cover 2,747 km2 in the Serengeti-Mara ecosystem, including most parts of the Masai Mara National Reserve and the northernmost section of the Serengeti National Park.<br>We chose these image acquisition dates and the spatial coverage according to the availability of the archived images and the requirements of the research goals: 1) to investigate whether the framework can be used to locate and count large herds of migratory ungulates accurately and automatically across a large and heterogeneous area in the Serengeti-Mara ecosystem using fine-resolution satellite imagery, 2) to quantify the variation in the wildebeest aggregation patterns across space and time 3) to examine if our approach works across different satellite sensors with various spatial resolutions. To fulfill these objectives, we chose the images in the dry-season range (from mid-July to mid-October) in the Masai Mara National Reserve and the northern part of the Serengeti National Park, where migratory wildebeest are expected to be highly aggregated and more cloud-free satellite images are likely to be available. Based on these requirements, we first preview the fine-resolution satellite imagery available on Google Earth, where we can visually check if the large herds of migratory ungulates are present. Then we further check the spatial resolution of these images at https://discover.maxar.com/ (a website for searching and discovering the archived high-resolution satellite images collected by Maxar satellite constellation) and select the images for this study.
Data exclusions	No data were excluded from the analyses.
Reproducibility	We repeated the same model training process with the same dataset 5 times and all attempts to repeat the experiment were successful.
Randomization	For model training, we selected the samples according to the complexity of environmental conditions and wildebeest patterns so that the model learns the diversity of the features. All the samples were grouped together for training. During training, the samples were split randomly into 10 folds for ensemble learning. This process is completely random.<br>For model testing, we selected the test image patches randomly within each strata using a stratified random sampling method. The strata are based on the number of animals in the image patches. The samples contain image patches with different levels of animal density (from 0 to ~800 animals on 336 by 336 pixels). Specifically, we use the model to detect and count the wildebeest on the images and estimate the wildebeest density in all sampling grids. The grid-level wildebeest density was used as the criteria to classify the grid cells into one of four categories (low density, medium density, high density and very high density) based on the mean and standard deviations. We then selected a proportionate number of samples randomly within each category to build the test dataset.
Blinding	The test dataset was sampled outside of the training areas and thus the model was evaluated on unseen data.

Did the study involve field work? ☐ Yes ☒ No

Reporting for specific materials, systems and methods

We require information from authors about some types of materials, experimental systems and methods used in many studies. Here, indicate whether each material, system or method listed is relevant to your study. If you are not sure if a list item applies to your research, read the appropriate section before selecting a response.

Materials & experimental systems

n/a	Involved in the study
☒	☐ Antibodies
☒	☐ Eukaryotic cell lines
☒	☐ Palaeontology and archaeology
☒	☐ Animals and other organisms
☒	☐ Clinical data
☒	☐ Dual use research of concern

Methods

n/a	Involved in the study
☒	☐ ChIP-seq
☒	☐ Flow cytometry
☒	☐ MRI-based neuroimaging
